# Genome‐wide association study discovered genetic variation and candidate genes of fibre quality traits in *Gossypium hirsutum* L.

**DOI:** 10.1111/pbi.12693

**Published:** 2017-03-07

**Authors:** Zhengwen Sun, Xingfen Wang, Zhengwen Liu, Qishen Gu, Yan Zhang, Zhikun Li, Huifeng Ke, Jun Yang, Jinhua Wu, Liqiang Wu, Guiyin Zhang, Caiying Zhang, Zhiying Ma

**Affiliations:** ^1^ North China Key Laboratory for Crop Germplasm Resources of Education Ministry/Key Laboratory for Crop Germplasm Resources of Hebei Province Hebei Agricultural University Baoding China

**Keywords:** *Gossypium hirsutum*, Genome‐wide association study, fibre quality, single nucleotide polymorphism, RNA‐sequencing

## Abstract

Genetic improvement of fibre quality is one of the main breeding goals for the upland cotton, *Gossypium hirsutum*, but there are difficulties with precise selection of traits. Therefore, it is important to improve the understanding of the genetic basis of phenotypic variation. In this study, we conducted phenotyping and genetic variation analyses of 719 diverse accessions of upland cotton based on multiple environment tests and a recently developed Cotton 63K Illumina Infinium SNP array and performed a genome‐wide association study (GWAS) of fibre quality traits. A total of 10 511 polymorphic SNPs distributed in 26 chromosomes were screened across the cotton germplasms, and forty‐six significant SNPs associated with five fibre quality traits were detected. These significant SNPs were scattered over 15 chromosomes and were involved in 612 unique candidate genes, many related to polysaccharide biosynthesis, signal transduction and protein translocation. Two major haplotypes for fibre length and strength were identified on chromosomes Dt11 and At07. Furthermore, by combining GWAS and transcriptome analysis, we identified 163 and 120 fibre developmental genes related to length and strength, respectively, of which a number of novel genes and 19 promising genes were screened. These results provide new insight into the genetic basis of fibre quality in *G. hirsutum* and provide candidate SNPs and genes to accelerate the improvement of upland cotton.

## Introduction

Cotton is one of the most important natural textile fibre crops and is extensively planted throughout the world (Chen *et al*., 2007; Wendel, 1989). The cotton genus, *Gossypium*, consists of approximately 46 diploid species and six allotetraploid species (Grover *et al*., 2014; Wendel and Cronn, 2003). Of these, *Gossypium hirsutum*, also known as ‘upland cotton’, is the most widely cultivated species, constituting more than 95% of the world's cotton production. Upland cotton is characterized by wide adaptability and high production. However, its low‐quality fibre requires improvement to meet human demands and progress in spinning technology.

The fibre quality of cotton is a complex quantitative trait that is controlled by multiple genes and is susceptible to environmental conditions (Paterson *et al*., 2003). The major fibre quality characteristics include length, strength, fineness (also called micronaire), uniformity and elongation, among which length, strength and fineness are most important for spinning yarn quality. Previous studies have dissected the genetic architecture of fibre quality through traditional quantitative trait locus (QTL) linkage mapping using biparental populations (Jamshed *et al*., 2016; Paterson *et al*., 2003; Shen *et al*., 2005; Yang *et al*., 2014b; Zhang *et al*., 2011). Many QTLs of fibre quality traits have been identified (Lacape *et al*., 2010; Said *et al*., 2013), providing significant insight into fibre genetics. However, most of these QTLs obtained from interspecific populations are not directly applicable to upland cotton improvement because they are localized in very large genetic regions and are often not stable across populations (Islam *et al*., 2016), and the molecular mechanisms underlying most of these QTLs are unknown.

Genome‐wide association study (GWAS) based on linkage disequilibrium (LD) can effectively associate genotypes with phenotypes in natural populations and simultaneously detect many natural allelic variations and candidate genes in a single study, in contrast to QTL linkage mapping (Gupta *et al*., 2005; Huang and Han, 2014; Nuzhdin *et al*., 2012). Because of its advantages, including high resolution, cost efficiency and nonessential pedigrees, GWAS has been applied to many important and complicated phenotypes in crops such as rice (Huang *et al*., 2010; Yano *et al*., 2016; Zhao *et al*., 2011), maize (Li *et al*., 2013; Thornsberry *et al*., 2001; Yang *et al*., 2014a), soybean (Han *et al*., 2015; Zhou *et al*., 2015b), sorghum (Mace *et al*., 2013; Morris *et al*., 2013) and millet (Jia *et al*., 2013). The identification and characterization of the genes associated with important agronomic traits is essential for understanding the genetic basis of phenotypic variation and for promoting crop improvement.

The first commercial high‐density CottonSNP63K array was recently developed; it provides a new resource for the genetic dissection of agronomically and economically important traits in cotton improvement (Hulse‐Kemp *et al*., 2015). Additionally, the release of the upland cotton TM‐1 genome sequence (Li *et al*., 2015; Zhang *et al*., 2015b) and the development of high‐throughput single nucleotide polymorphism (SNP) assays (Zhao *et al*., 2011) have enabled GWAS to explore the genetic basis of complex cotton traits. However, there are few reports of GWA mapping using large natural populations based on SNP markers in cotton.

Thus, in this study, to identify the natural allelic variation in the cotton genome and candidate genes significantly associated with fibre quality, we performed a GWAS of fibre quality using 719 upland cotton accessions and the high‐density CottonSNP63K array based on phenotypic tests in eight environments. The results further our understanding of the mechanisms underpinning fibre quality, provide molecular markers for high‐quality cotton breeding and may provide a reference for genetic dissection of other complex quantitative traits in cotton.

## Results

### Phenotypic variation in fibre quality traits

To evaluate the phenotypic variation in fibre quality traits in the association population, we analysed five traits in eight environments during two years. Fibre length (FL), fibre strength (FS), fibre micronaire (FM), fibre uniformity (FU) and fibre elongation (FE) varied from 22.07 to 35.56 mm, 22.69 to 36.80 cN/tex, 3.14 to 6.34, 77.48 to 89.00 % and 3.55 to 8.65 %, respectively (Table 1). The combined variance analysis based on phenotype traits in the eight environments showed significant differences among genotypes, environments and a genotype and environment interaction (Table S2). Correlation analysis of the traits showed that FL was significantly positively correlated with FS and FU, and a positive correlation was also found between FS and FU. FM was significantly negatively correlated with FL and FS (Figure S1).

**Table 1:** Phenotypic variation for five fibre quality traits in the association population

Trait	Environment	Mean	SD	Max	Min	CV(%)
FL(mm)	14BD	29.27	1.73	33.68	22.07	5.92
	14HJ	28.76	1.43	34.78	23.91	4.97
	14XJ	30.13	1.41	35.20	22.73	4.69
	14QX	29.52	1.45	35.56	24.50	4.91
	14HN	29.25	1.25	33.60	24.50	4.27
	15XJ	30.02	1.29	35.14	22.74	4.28
	15QX	26.69	1.24	31.08	23.75	4.66
	15HN	29.06	1.43	34.80	23.17	4.91
FS(cN/tex)	14BD	29.40	2.45	35.80	23.30	8.32
	14HJ	29.10	2.21	35.00	23.30	7.59
	14XJ	30.82	2.13	36.30	25.20	6.92
	14QX	30.09	2.14	36.80	24.70	7.10
	14HN	28.36	2.11	36.30	23.10	7.44
	15XJ	31.03	1.84	36.61	25.85	5.94
	15QX	28.43	1.99	34.16	23.24	7.01
	15HN	25.63	1.78	32.05	22.69	6.95
FM	14BD	5.10	0.46	6.34	3.50	8.97
	14HJ	5.21	0.40	6.21	3.67	7.72
	14XJ	5.07	0.42	6.20	3.14	8.24
	14QX	5.00	0.39	6.03	3.52	7.88
	14HN	4.50	0.47	5.78	3.23	10.47
	15XJ	4.80	0.34	5.65	3.93	7.04
	15QX	5.36	0.28	6.15	4.10	5.31
	15HN	4.58	0.43	6.16	3.16	9.31
FU (%)	14BD	84.99	1.25	88.00	80.60	1.47
	14HJ	85.09	1.25	89.00	80.40	1.46
	14XJ	85.12	1.10	88.10	80.80	1.30
	14QX	84.72	1.18	88.30	78.90	1.40
	14HN	85.28	0.98	88.30	82.25	1.15
	15XJ	85.34	0.99	88.29	81.31	1.16
	15QX	82.76	1.21	86.01	77.48	1.46
	15HN	85.72	0.96	88.40	82.75	1.12
FE (%)	14BD	6.75	0.07	7.00	6.50	1.05
	14HJ	6.75	0.06	6.90	6.50	0.94
	14XJ	6.79	0.06	7.00	6.60	0.83
	14QX	6.78	0.06	7.00	6.50	0.93
	14HN	5.91	0.88	8.65	3.55	14.89
	15XJ	7.19	0.24	8.06	6.47	3.29
	15QX	6.35	0.26	7.23	5.72	4.07
	15HN	6.66	0.24	8.25	5.97	3.65

FL, fibre length; FS, fibre strength; FM, fibre micronaire; FU, fibre uniformity; FE, fibre elongation; SD, standard deviation, which was calculated based on the measured values of the fibre traits from two replicates in 2014 and three replicates in 2015; CV, coefficient of variation.

### Genetic variation based on SNPs

**Figure 1:**
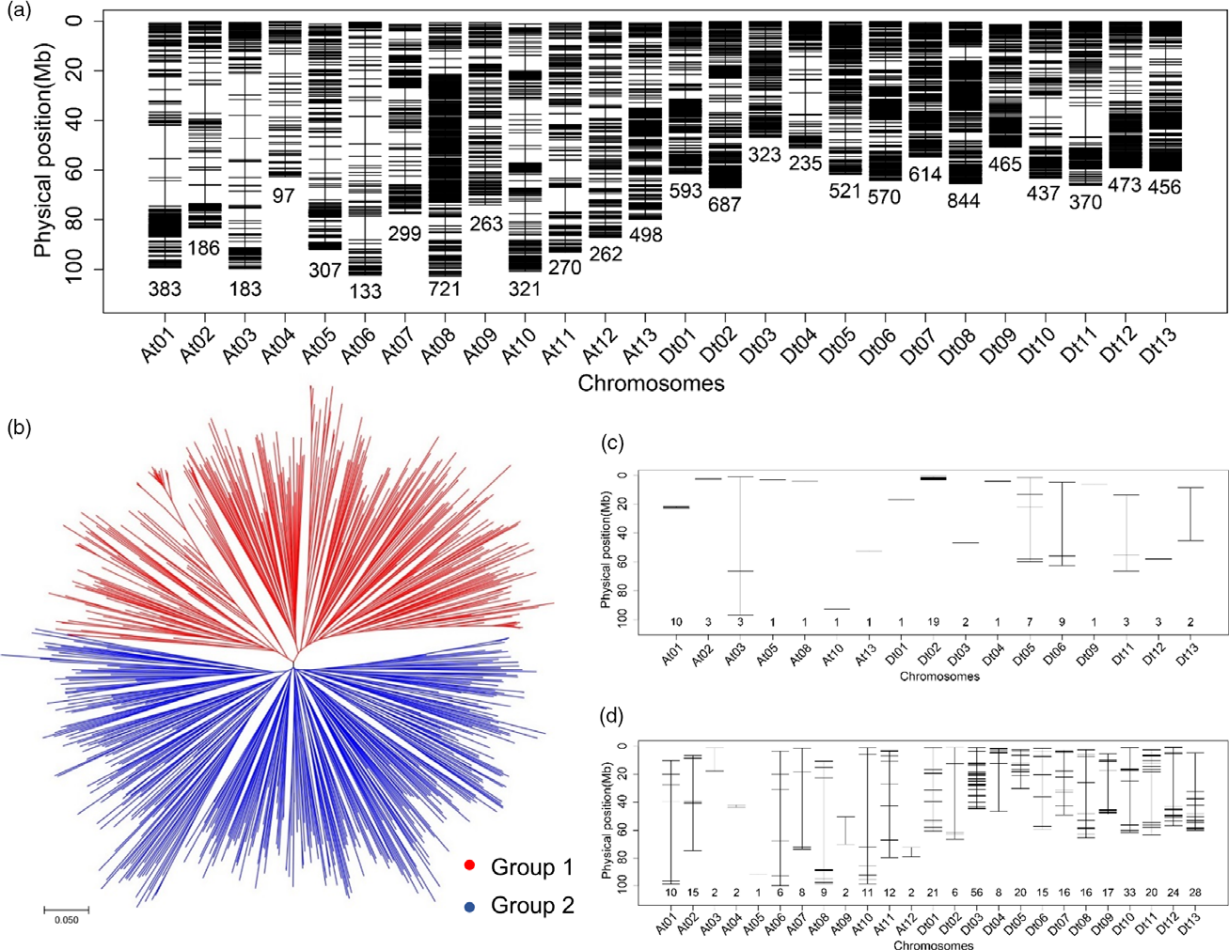
Genetic divergence of upland cotton germplasms. (a) Genome‐wide SNP density in the entire association mapping panel. Dark and white horizontal bars show genomic regions that are rich and poor in SNPs, respectively. (b) Neighbour‐joining tree of all cotton accessions in this study. Accessions in the NJ tree are represented by different colours: Group 1 (red) and Group 2 (blue). (c) Distribution of SNPs that only showed polymorphism in Group 1. (d) Distribution of SNPs that only showed polymorphism in Group 2. The number of SNP markers anchored to different chromosomes of the *G. hirsutum* genome is given on the horizontal axis.

The genotypes of 719 accessions were examined using the Cotton 63K Illumina Infinium SNP array. All the SNP data were analysed using Illumina GenomeStudio software. First, SNPs without allele polymorphism were eliminated. Low‐quality SNP loci (call rate < 85% and minor allele frequency < 0.05) were also deleted. A final set of 10 511 high‐quality SNPs was screened and used for genetic variation and GWA analysis (Figure 1a; Table 2). These SNP markers were not evenly distributed across the entire genome, with 3923 and 6588 SNPs in the At and Dt subgenomes, respectively. Chromosome Dt08 had the maximum number of SNPs (844), and At04 had the minimum (97) (Figure 1a; Table 2). The polymorphism information content (PIC) values ranged from 0.208 to 0.312 among chromosomes, and the mean PIC of the At and Dt subgenomes was 0.287 and 0.283, respectively (Table 2).

**Table 2:** The summary of the number of polymorphic SNPs mapped in 26 chromosomes of *Gossypium hirsutum*

Chr.	No. of SNPs	Chr. Size (Mb)	Density of SNP (kb/SNP)	PIC
At01	383	99.9	260.8	0.310
At02	186	83.5	448.7	0.288
At03	183	100.3	547.9	0.294
At04	97	62.9	648.6	0.312
At05	307	92.1	299.8	0.307
At06	133	103.2	776.2	0.277
At07	299	78.3	261.7	0.300
At08	721	103.7	143.8	0.208
At09	263	75.0	285.2	0.308
At10	321	100.9	314.2	0.272
At11	270	93.3	345.6	0.246
At12	262	87.5	333.9	0.296
At13	498	80.0	160.6	0.308
Dt01	593	61.5	103.6	0.278
Dt02	687	67.3	97.9	0.311
Dt03	323	46.7	144.6	0.241
Dt04	235	51.5	218.9	0.283
Dt05	521	61.9	118.9	0.299
Dt06	570	64.3	112.8	0.247
Dt07	614	55.3	90.1	0.294
Dt08	844	61.9	73.4	0.277
Dt09	465	51.0	109.7	0.293
Dt10	437	63.4	145.0	0.295
Dt11	370	66.1	178.6	0.282
Dt12	473	59.1	125.0	0.293
Dt13	456	60.5	132.7	0.285

Chr., chromosome; PIC, polymorphism information content

To understand the genetic diversity of our association panel, molecular phylogenetic analysis among these accessions was conducted based on the genetic distances of these SNPs. The neighbour‐joining (NJ) tree results showed two divergent groups (Figure 1b), designated G1 and G2, with 323 and 396 accessions, respectively (Table S1). We then compared the differences in genome‐wide SNPs between the two groups. There were 360 unique SNPs across the accessions of G2 and only 68 unique SNPs in G1 (Figure 1c, d). These results suggested the two groups had a degree of genetic differentiation at the molecular level (Coart *et al*., 2002).

### Population structure and linkage disequilibrium

**Figure 2:**
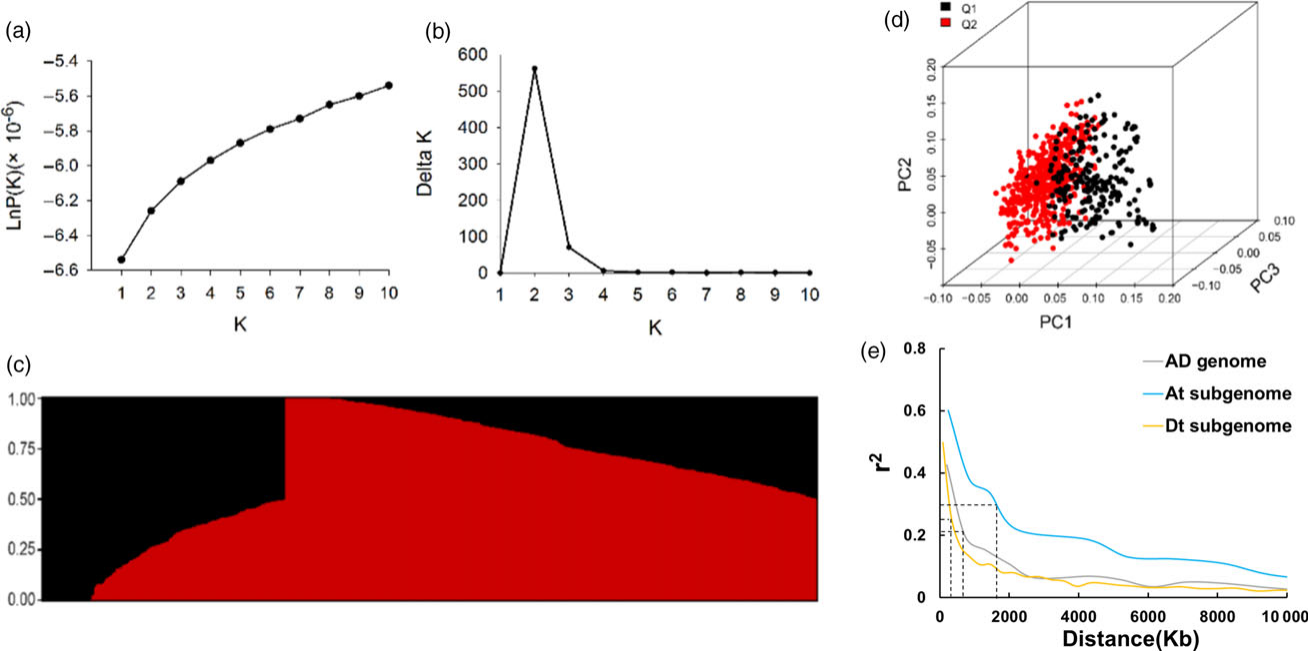
Analysis of the population structure of 719 upland cotton accessions. (a) Estimated LnP(K) of possible clusters (K) from 1 to 10. (b) Delta K based on the rate of change of LnP(K) between successive K. (c) Population structure based on STRUCTURE when K = 2. (d) Principal component analysis showing the population structure in the diversity panel. Two subpopulations are designated Q1 (black) and Q2 (red). (e) Genome‐wide average LD decay estimated in the AD genome (grey), At subgenome (blue) and Dt subgenome (yellow).

We investigated the population structure of our panel using STRUCTURE software. The LnP(K) values continuously increased with K from 1 to 10 (Figure 2a); however, the delta K value reached a sharp peak at K = 2 (Figure 2b). Therefore, this association population was clustered into two subpopulations (Figure 2c). Similarly, PCA showed two clusters for the population, despite some accessions overlapping in the two clusters (Figure 2d). Combined with the results of the normal distribution of all investigated traits (Figure S1), the varietal population in this study was considered not highly structured and could be used for GWAS as in other previously reported crops (Yano *et al*., 2016). The extent of LD provides a moderate resolution for genome‐wide identification for gene discovery (Flint‐Garcia *et al*., 2003). The LD decay of our population was approximately 0.82 Mb, where the *r* ^2^ drops to half the maximum value (Figure 2e). The overall LD decay distance in the At subgenome was significantly higher than that in the Dt subgenome, 1.30 and 0.33 Mb, respectively (Figure 2e). Considering the LD decay distances and in comparison with other crops, such as rice and maize, with LD of 100 kb‐1 Mb and 1‐100 kb, respectively (Gore *et al*., 2009; Nordborg *et al*., 2002; Remington *et al*., 2001; Zhao *et al*., 2011), we assumed 200 kb as the region of SNP‐associated candidate genes for fibre traits.

### Genome‐wide association mapping

To explore the genetic factors associated with the fibre quality, we conducted a GWAS, which took into account the population structure and familial relatedness (Yu et al., 2006) using all traits analysed in 2014 and 2015. A total of 46 significantly associated SNPs were detected in at least one environment (Table 3). These SNPs were located on chromosomes At01, At07, At08, At10, At12, At13, Dt01, Dt03, Dt04, Dt05, Dt06, Dt07, Dt10, Dt11 and Dt13. In addition, some were detected repeatedly across multiple environments, which was similar to the results for other plants, showing more stable SNPs linked with traits (Xu *et al*., 2016; Zhao *et al*., 2011).

**Table 3:** The summary of SNPs significantly associated with fibre quality traits

Traits	SNPs	Chr.	Site	Allele	MAF	‐Log_10_(P)	R^2^(%)	Environment
FL	i39753Gh	At07	72067994	G/A	0.09(G)	4.78	3.32	E4
	i02033Gh	At07	72193182	G/T	0.12(G)	4.05–4.15	2.70–2.73	E4,E5
	i02034Gh	At07	72198802	A/G	0.07(A)	5.04	3.28	E4
	i02035Gh	At07	72200974	C/T	0.07(C)	5.06	3.30	E4
	i02037Gh	At07	72204773	C/T	0.07(C)	5.04	3.28	E4
	i49171Gh	At07	72213592	T/C	0.11(T)	4.09	2.66	E4
	i37604Gh	At07	72249786	C/T	0.11(C)	4.09	2.66	E4
	i12279Gh	At10	98329643	A/G	0.38(A)	4.57	2.98	E3
	i24710Gh	At10	98399227	A/G	0.30(A)	4.55	2.99	E3
	i23677Gh	At10	98423067	T/C	0.30(T)	4.55	2.99	E3
	i03207Gh	Dt03	3409179	G/A	0.05(G)	3.98	2.60	E8
	i09441Gh	Dt05	12903672	A/G	0.34(A)	3.97–4.33	2.58–2.83	E2,E4,E8
	i34936Gh	Dt05	13762535	T/G	0.31(T)	4.69	3.07	E5
	i49170Gh	Dt06	174910	G/A	0.37(G)	4.66	3.03	E4
	i44154Gh	Dt07	42826098	C/A	0.13(C)	3.99	2.59	E4
	i44994Gh	Dt07	42837391	G/A	0.13(G)	3.99	2.59	E4
	i07326Gh	Dt11	23906867	C/T	0.27(C)	4.41	2.91	E1
	i22531Gh	Dt11	23959318	A/G	0.27(A)	4.41	2.91	E1
	i07327Gh	Dt11	24008823	T/C	0.27(T)	4.41	2.91	E1
	i60962Gt	Dt11	24030081	A/G	0.18(A)	4.32–7.26	2.12–4.78	E1,E2,E4‐E6,E8
FS	i65738Gm	At01	96310264	A/C	0.07(A)	4.11	2.69	E1
	i18340Gh	At07	71993462	C/A	0.12(C)	5.17	3.38	E4
	i44206Gh	At07	72008085	G/A	0.12(G)	5.17	3.38	E4
	i39753Gh	At07	72067994	G/A	0.09(G)	4.27–7.56	3.22–5.38	E1‐E4
	i02033Gh	At07	72193182	G/T	0.12(G)	5.29	3.53	E4
	i02034Gh	At07	72198802	A/G	0.07(A)	4.04–9.16	2.81–6.07	E1‐E4
	i02035Gh	At07	72200974	C/T	0.07(C)	4.03–9.15	2.80–6.07	E1‐E4
	i02037Gh	At07	72204773	C/T	0.07(C)	4.04–9.16	2.81–6.07	E1‐E4
	i49171Gh	At07	72213592	T/C	0.11(T)	5.32	3.48	E4
	i37604Gh	At07	72249786	C/T	0.11(C)	5.32	3.48	E4
	i30934Gh	At13	5168143	T/C	0.30(T)	4.59	2.97	E7
	i38606Gh	Dt06	60561982	T/C	0.08(T)	4.16	2.72	E5
	i22089Gh	Dt06	60565551	A/G	0.08(A)	4.16	2.72	E5
	i20058Gh	Dt10	23109967	T/C	0.10(T)	4.32	2.79	E7
	i60962Gt	Dt11	24030081	A/G	0.18(A)	4.50	2.94	E8
	i41872Gh	Dt13	52825264	G/T	0.27(G)	4.26	2.96	E2
	i09756Gh	Dt13	52919060	T/C	0.34(T)	4.18	2.73	E4
	i20350Gh	Dt13	52934633	C/T	0.35(C)	4.32	2.93	E6
FM	i49257Gh	At13	13057186	G/A	0.45(G)	4.02	2.57	E7
	i45804Gh	At13	13494588	A/G	0.45(A)	4.38	2.81	E7
	i25107Gh	Dt05	60697515	C/T	0.49(C)	4.33	2.90	E5
	i46147Gh	Dt06	2045744	C/T	0.47(C)	4.61	2.96	E7
FU	i04566Gh	At08	97378358	G/A	0.47(G)	4.90	3.19	E3
	i15200Gh	At08	97451057	T/C	0.49(T)	4.39	2.85	E3
	i04572Gh	At08	97452659	G/A	0.49(G)	4.27	2.77	E3
	i54149 Gb	At08	97482110	G/A	0.49(G)	4.48	2.91	E3
FE	i24710Gh	At10	98399227	A/G	0.30(A)	4.18	2.67	E3
	i23677Gh	At10	98423067	T/C	0.30(T)	4.18	2.67	E3
	i27874Gh	At12	2806482	C/T	0.20(C)	4.28	2.80	E5
	i24077Gh	At12	2849310	G/T	0.20(G)	4.33	2.83	E5
	i16244Gh	At12	2885883	C/T	0.21(C)	4.09	2.67	E5
	i24987Gh	Dt01	39191073	A/G	0.05(A)	5.04	3.28	E8
	i21001Gh	Dt01	39365919	A/G	0.05(A)	5.04	3.28	E8
	i51597 Gb	Dt03	425957	C/T	0.37(C)	4.12	2.59	E6
	i12839Gh	Dt04	47872770	A/C	0.32(A)	8.23	5.45	E5
	i12840Gh	Dt04	47872954	T/G	0.32(T)	7.83	5.18	E5
	i09441Gh	Dt05	12903672	A/G	0.34(A)	4.16	2.68	E2

FL, fibre length; FS, fibre strength; FM, fibre micronaire; FU, fibre uniformity; FE, fibre elongation; ‐Log_10_(P) value indicates the significance levels and R^2^ (%) indicates the percentage of phenotypic variation explained by each SNP; Chr., chromosome; MAF, minor allele frequency.

For FL, 20 significant SNPs were identified on chromosomes At07, At10, Dt03, Dt05, Dt06, Dt07 and Dt11 (Table 3; Figure S2), explaining approximately 57.72 % of the total phenotypic variation (Table 3). Seven loci in At07 and four loci in Dt11 were close in their respective chromosomes. Moreover, the locus i60962Gt in Dt11 was detected across six environments (Table 3).

For FS, 18 significant SNPs on chromosomes At01, At07, At13, Dt06, Dt10, Dt11 and Dt13 were detected (Table 3; Figure S3), contributing 2.69 to 6.07 % of the total phenotypic variation (Table 3). Among these SNPs, four loci were detected repeatedly in four environments (Table 3; Figure S4). Seven SNPs in At07 were also obtained from loci related to FL (Table 3).

For the other three traits, FM, FU and FE, 4, 4 and 11 loci were detected, respectively (Table 3). There were four SNPs located in three chromosomes significantly associated with FM (Table 3; Figure S4), and all of four loci for FU were located close together in At08 (Table 3; Figure S5). The 11 SNPs for FE on six chromosomes contributed 2.67 to 5.45 % of the phenotypic variation, but three (i24710Gh, i23677Gh and i09441Gh) were also present among significant SNPs associated with FL (Table 3; Figure S6).

### Identification of candidate genes associated with SNPs

**Figure 3:**
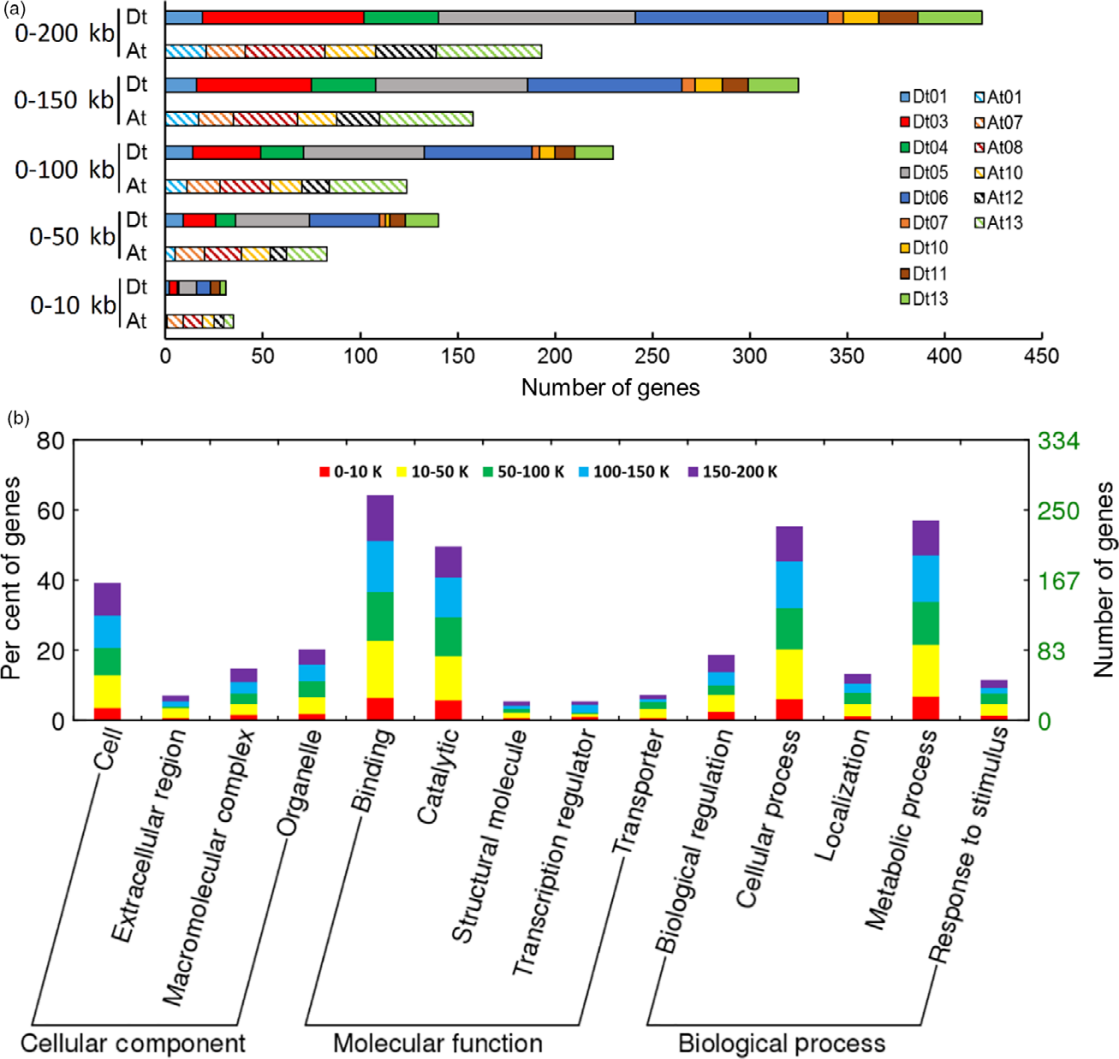
Chromosome distribution and gene ontology (GO) analysis of 612 candidate genes in five consecutive intervals and their distance to the nearest associated SNP. (a) Gene numbers in different chromosome of the At subgenome and Dt subgenome. (b) Functional classification of all genes in three levels: cellular component, molecular function and biological process. The five colours represent five different intervals.

We confirmed potential candidate genes near 46 significant SNP loci based on genes annotated in the *G. hirsutum* TM‐1 genome (Zhang *et al*., 2015b). Within 200 kb of significant SNPs, a total of 612 candidate genes were identified (Table S3). We scrutinized the distribution of these candidate genes among each chromosome according to the distance to the nearest associated SNP (Figure 3a; Table S3). The results showed that genes detected in the Dt subgenome overall doubled those in the At subgenome (Figure 3a). The genes on Dt03, Dt05 and Dt06 increased as the distance increased, and genes on At07, At10 and Dt11 showed no obvious change (Figure 3a).

Gene ontology (GO) analysis indicated that the functions of most genes were binding and catalytic reactions, regardless of the intervals (Figure 3b). We also conducted KEGG pathway enrichment of all candidate genes, and found 284 genes enriched in 72 pathways, with approximately 40 genes involved in important fibre development pathways (Table S4). The top three concrete pathways containing more than ten genes were plant hormone signal transduction, carbon metabolism and ribosome pathways. For the nucleotide sugar metabolism and starch and sucrose metabolism pathways clearly related to fibre development, two genes, *Gh_A13G0573* and *Gh_D07G1799*, code for galacturonosyltransferase (GAUT), and *Gh_A07G1759* and *Gh_D06G1953* code for ADP glucose pyrophosphorylase and UDP‐arabinose 4‐epimerase, respectively (Tables S3, S4). We analyse these genes and their related transcriptome data below.

### Analysis of major SNP loci relevant to fibre length and strength

**Figure 4:**
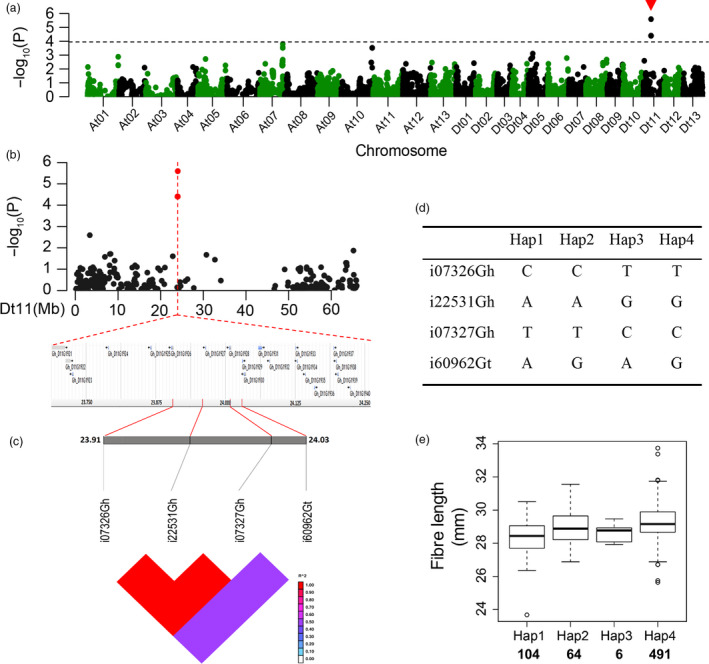
GWAS results for fibre length and analysis of the peak on chromosome Dt11. (a) Manhattan plots for fibre length. The dashed line represents the significance threshold (*P *< 10^−3.97^). The arrowhead indicates the position of the strong peak investigated in this study. (b) Manhattan plot (top) and genes surrounding the peak (bottom) on chromosome Dt11. (c) Genomic location of four SNP loci and LD based on pairwise R^2^ values between the SNPs estimated in Dt11. The R^2^ values are indicated using the colour bar. (d) Haplotypes observed in 719 accessions using the four SNPs. (e) Phenotypic differences of fibre length among four haplotypes.

We analysed major SNP loci relevant to the two critical quality traits, fibre length and strength, that breeders most often consider in selection. For SNPs associated with FL, a significant peak appeared in chromosome Dt11 (Figure 4a, b), and there were 20 candidate genes associated with the significant SNPs (Figure 4b). Haplotype analysis showed a high level of LD between the associated SNPs in Dt11 (Figure 4c). The four SNPs resulted in four haplotypes in our association panel (Figure 4d). The average FL of Hap4 was 29.24 mm, greater than those of the other three (Figure 4e). Based on the polymorphism of the SNP markers, each SNP locus of Hap4 could be classified into three genotypes (Figure S7). The genotypes of the four SNP loci containing in Hap4 displayed a higher average FL than the other nonincluded genotypes (Figure S7).

**Figure 5:**
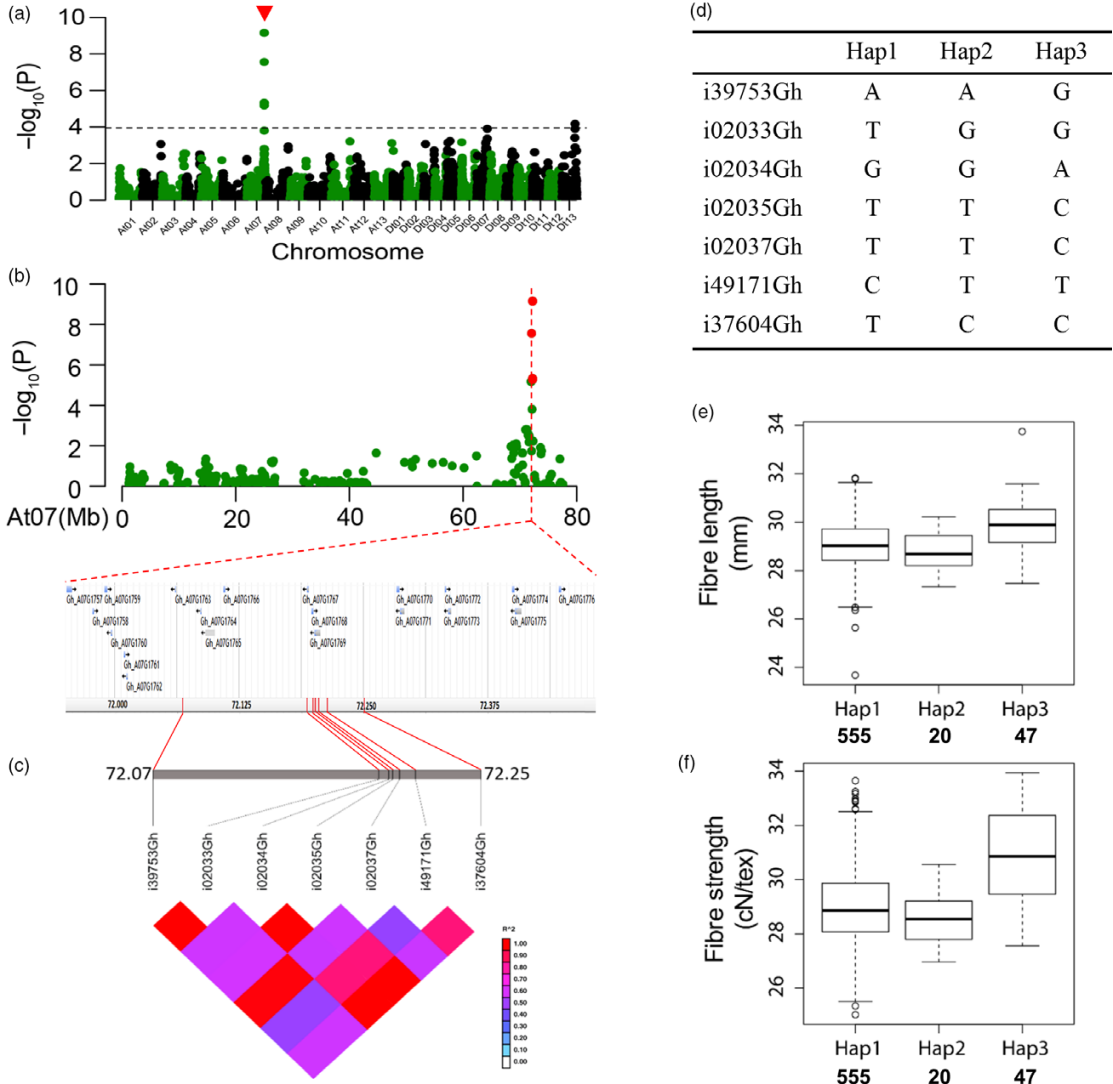
GWAS results for fibre strength and analysis of the peak on chromosome At07. (a) Manhattan plots for fibre strength. The dashed line represents the significance threshold (*P *< 10^−3.97^). The arrowhead indicates the position of the strong peak investigated in this study. (b) Manhattan plot (top) and genes surrounding the peak on chromosome At07 (bottom). (c) Genomic location of seven SNP loci and LD based on the pairwise R^2^ values between the SNPs estimated in At07. The R^2^ values are indicated using the colour bar. (d) Haplotypes observed in 719 accessions using the seven SNPs. (e‐f) Phenotypic differences of fibre length and strength among three haplotypes.

Seven significant SNPs on chromosome At07 were simultaneously analysed for fibre length and strength across multiple environments (Table 3; Figure 5a, b). We selected seven SNPs to further investigate the allelic variation. There were also 20 candidate genes surrounding them (Figure 5b). Haplotype analysis showed a high level of LD between these SNP markers (Figure 5c). Among the 719 accessions, there were three haplotypes with the seven SNPs (Figure 5d). An overwhelming majority of the accessions belonged to Hap1, while Hap3 was composed of 47 accessions (Table S5), some of which were known high‐quality upland cotton cultivars. The average fibre length and strength of Hap3 were 29.85 mm and 30.81 cN/tex, respectively, significantly greater than those of other two (Figure 5e, f).

For the genetic effect of each SNP in Hap3, taking locus i39753Gh for example, the average fibre length and strength in the accessions with the GG genotype were 29.90 mm and 31.37 cN/tex, respectively (Figure S8). The three SNPs i02034Gh, i02035Gh and i02037Gh had the same effect on the phenotype as the locus i39753Gh (Figure S8). Moreover, the genotype of the other loci in Hap3 had a higher average fibre length and strength than those of the other haplotypes (Figure S8).

### Verification of candidate genes by transcriptome analysis

**Figure 6:**
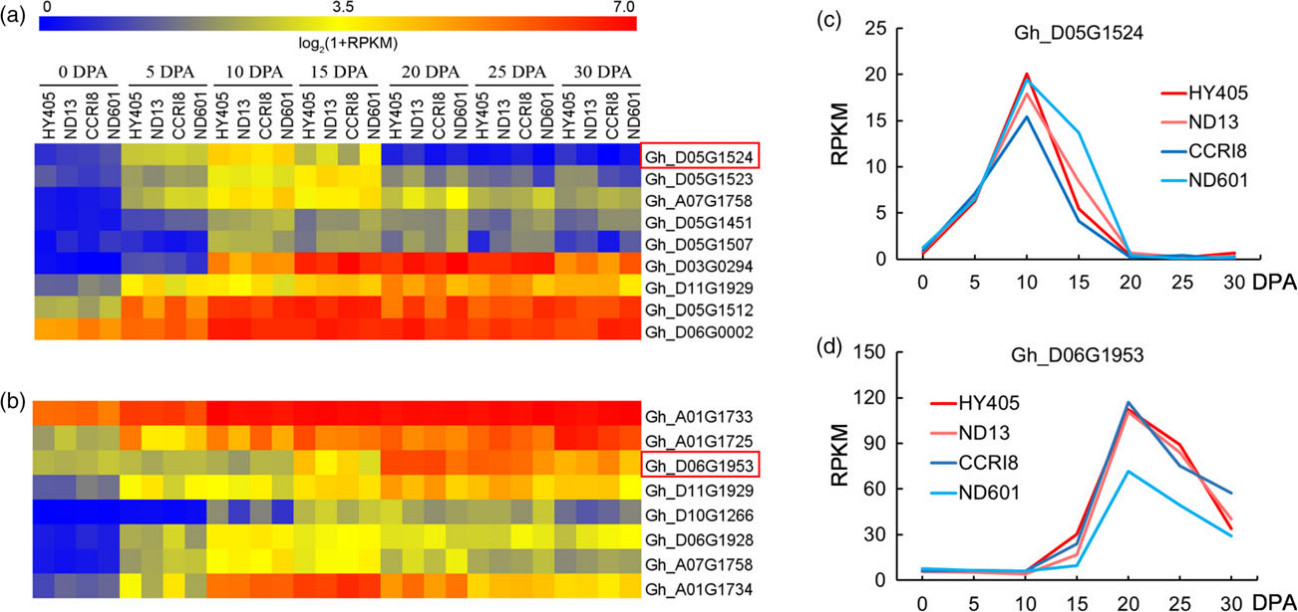
Expression pattern of the promising genes involved in fibre development in four upland cotton cultivars (HY405, ND13, CCRI8 and ND601). (a‐b) Heat map of the expression of genes related to fibre length and strength. (c‐d) Expression levels of two representative genes associated with fibre length and strength.

We further validated these candidate genes using transcriptome sequencing data. We investigated the expression level (log_2_(1+RPKM) > 1) of all 212 and 161 genes related to FL and FS, respectively (Table S3), and found that approximately two‐thirds of genes (163 and 120) were significantly expressed during some time points (0–30 days post anthesis, DPA) of fibre development, including initiation, cell elongation and cell wall thickening (Figure S9). Among them, there were 107 and 95 genes (including 30 and 27 hypothetical proteins) not reported in cotton (Table S3). This means the genes are novel candidates for fibre development. The genes that had been reported in cotton (Deng *et al*., 2016; Guo *et al*., 2009; Huang *et al*., 2013; Samuel *et al*., 2006; Wang *et al*., 2004; Zhou *et al*., 2015a) were mainly (63 % in FL and 68 % in FS) involved in fibre development, which confirmed the accuracy of the fibre‐associated candidate genes described in this study. The differences in fibre length and strength between cultivars could be determined by gene expression in different stages of fibre development. For genes in the fibre elongation stage (0–15 DPA), those with significantly increased expression levels in at least two of the three time points (5, 10 and 15 DPA) compared with 0 DPA (predominately expressed) were screened in four upland cotton cultivars. There were nine candidate genes that matched this condition (Figure 6a). In the fibre thickening stage, spanning 20–30 DPA, we identified eight differentially expressed genes (Figure 6b).

All 17 selected genes (nine in FL and eight in FS) were related to diverse functions (Table 4) and were classified into three expression patterns (Figure 6). First, some genes, *for example Gh_D05G1524*, encoding a lipid transfer protein, showed steeply increasing expression from 0 DPA of fibre elongation stage and sharply decreased after 10 DPA among four upland cotton cultivars (Figure 6c). We designated this type of gene ‘elongation pattern’. Second, another type of gene, *for example Gh_D06G1953*, associated with fibre length, coding for UDP‐arabinose 4‐epimerase in the nucleotide sugar metabolism pathway (Table S4) and catalysing the epimerization between UDP‐D‐Xyl and UDP‐L‐Ara in the cell wall polysaccharides (Burget *et al*., 2003), showed a striking increase in expression from 15 DPA, peaking at 20 DPA (Figure 6d). We designated this type of gene ‘thickening pattern’. Third, several of the 17 genes, such as *Gh_D06G0002* and *Gh_A01G1733*, were highly expressed from 5 to 30 DPA (Figure 6a, b). We designated this type of gene ‘elong‐ckening pattern’. Other than the five known genes (*Gh_D03G0294*,* Gh_D05G1451, Gh_D05G1507*,* Gh_D05G1523* and *Gh_D07G1799*), ten (excluding two genes found in both FL and FS) of these 17 predominately expressed genes were not previously reported in cotton (Table S3). Additionally, there were three expression patterns of fibre development for the 163 and 120 screened genes of FL and FS (Figure S9).

**Table 4:** Promising genes associated with fibre length and strength identified by GWAS combined with RNA‐seq

Trait	Gene ID	Homologue	Gene annotation	Distance (kb)
FL	Gh_A07G1758	AT4G17170.1	RAB GTPase homologue B1C	88.4
	Gh_D03G0294	AT5G65730.1	Xyloglucan endotransglucosylase/hydrolase 6	129.8
	Gh_D05G1451	AT1G78580.1	trehalose‐6‐phosphate synthase	21.1
	Gh_D05G1507	AT1G28480.1	Thioredoxin superfamily protein	190.5
	Gh_D05G1512	AT2G20560.1	DNAJ heat‐shock family protein	132.2
	Gh_D05G1523	AT5G49760.1	Leucine‐rich repeat protein kinase family protein	10.6
	Gh_D05G1524	AT5G49800.1	Polyketide cyclase/dehydrase and lipid transport superfamily protein	1.2
	Gh_D06G0002	AT1G47830.1	SNARE‐like superfamily protein	167.8
	Gh_D11G1929	AT3G19150.1	KIP‐related protein 6	123.2
	Gh_D07G1799	AT3G02350.1	galacturonosyltransferase 9	32.4
FS	Gh_A01G1725	AT3G11660.1	NDR1/HIN1‐like 1	98.5
	Gh_A01G1733	AT3G52560.1	ubiquitin E2 variant 1D‐4	26.9
	Gh_A01G1734	AT5G06270.1	unknown protein	52.7
	Gh_A07G1758	AT4G17170.1	RAB GTPase homologue B1C	88.4
	Gh_D06G1928	AT5G58380.1	CBL‐interacting protein kinase	153.2
	Gh_D06G1953	AT1G30620.2	UDP‐arabinose 4‐epimerase 1	155.0
	Gh_D10G1266	AT3G57880.1	CaLB domain plant phosphoribosyltransferase family protein	124.6
	Gh_D11G1929	AT3G19150.1	KIP‐related protein 6	123.2
	Gh_D13G1792	AT3G04470.1	Ankyrin repeat family protein	24.9

FL, fibre length; FS, fibre strength; FM, fibre micronaire; FU, fibre uniformity; FE, fibre elongation; Distance indicates the distance to the nearest SNP.

**Figure 7:**
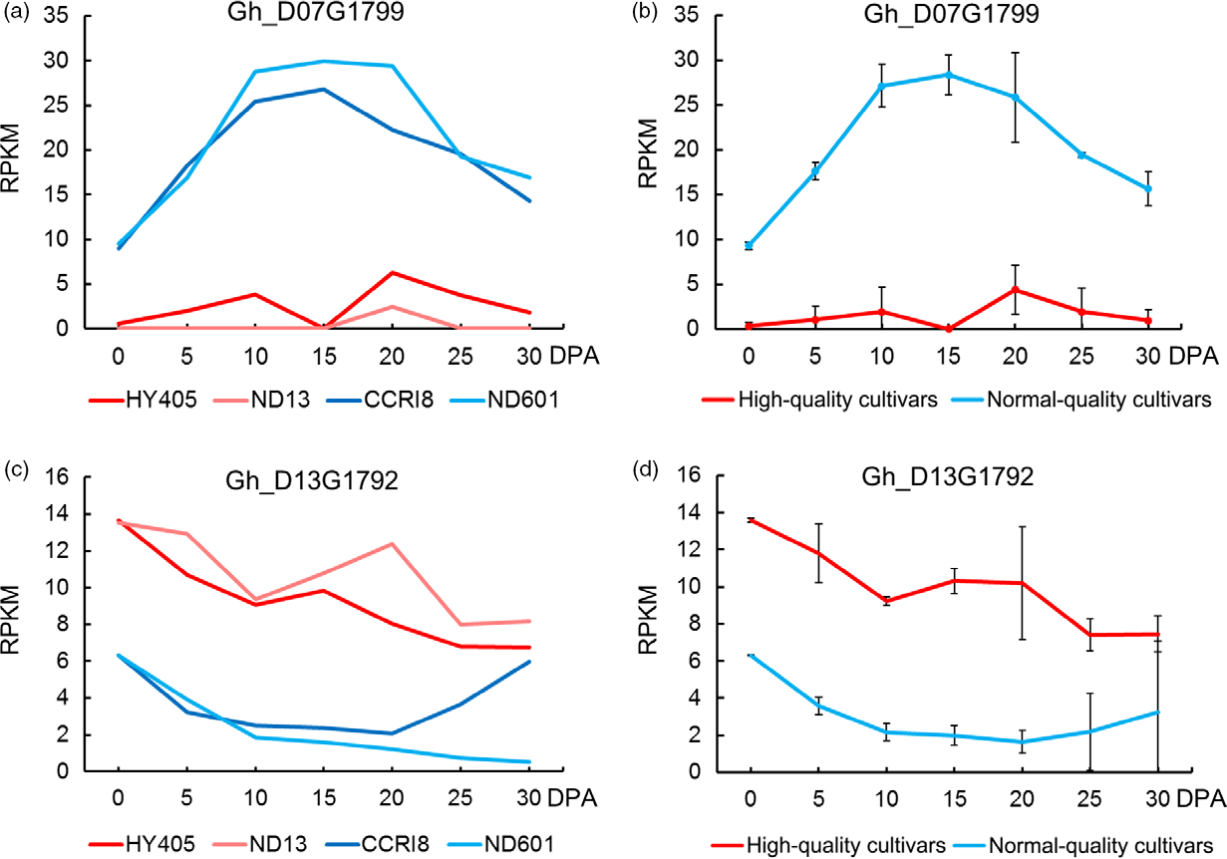
Expression levels of genes significantly differentially expressed between high‐quality cultivars (HY405 and ND13) and normal‐quality cultivars (CCRI8 and ND601). (a) Expression of the gene *Gh_D07G1799*, associated with fibre length, in the four cultivars. (b) Expression of the gene *Gh_D07G1799* between high‐quality cultivars and normal‐quality cultivars. (c) Expression of the gene *Gh_D13G1792*, associated with fibre strength, in the four cultivars. (d) Expression of the gene *Gh_D13G1792* between high‐quality cultivars and normal‐quality cultivars.

We found two genes with significant differences in expression between high‐quality and normal‐quality cultivars (Figure 7; Table 4). *Gh_D07G1799*, coding for galacturonosyltransferase (GAUT), is involved in pectin biosynthesis, which is part of glycan metabolism (Sterling *et al*., 2006). In the KEGG analysis, we found that it was from the nucleotide sugar metabolism and sucrose metabolism pathways (Table S4). In the previous study of our team, we cloned a novel gene, *GbGAUT1*, from *G. barbadense* and proposed that *GbGAUT1* plays a key role in fibre development (Chi *et al*., 2009). The expression level of *Gh_D07G1799* showed an obvious increase in fibre elongation stage and was expressed at much higher levels in normal‐quality cultivars than in high‐quality cultivars (Figure 7a, b). Another gene *Gh_D13G1792* belonged to the Arabidopsis ankyrin repeat family protein, and this protein served as a molecular chaperone that plays many important roles in plant cellular metabolism (Shen *et al*., 2010). It is associated with membrane‐enclosed organelles and is required for pollen tube growth in lilies (Huang *et al*., 2006). However, it was not previously reported in cotton (Table S3). This gene had a much higher expression level in high‐quality cultivars than in normal‐quality cultivars (Figure 7c, d).

**Figure 8:**
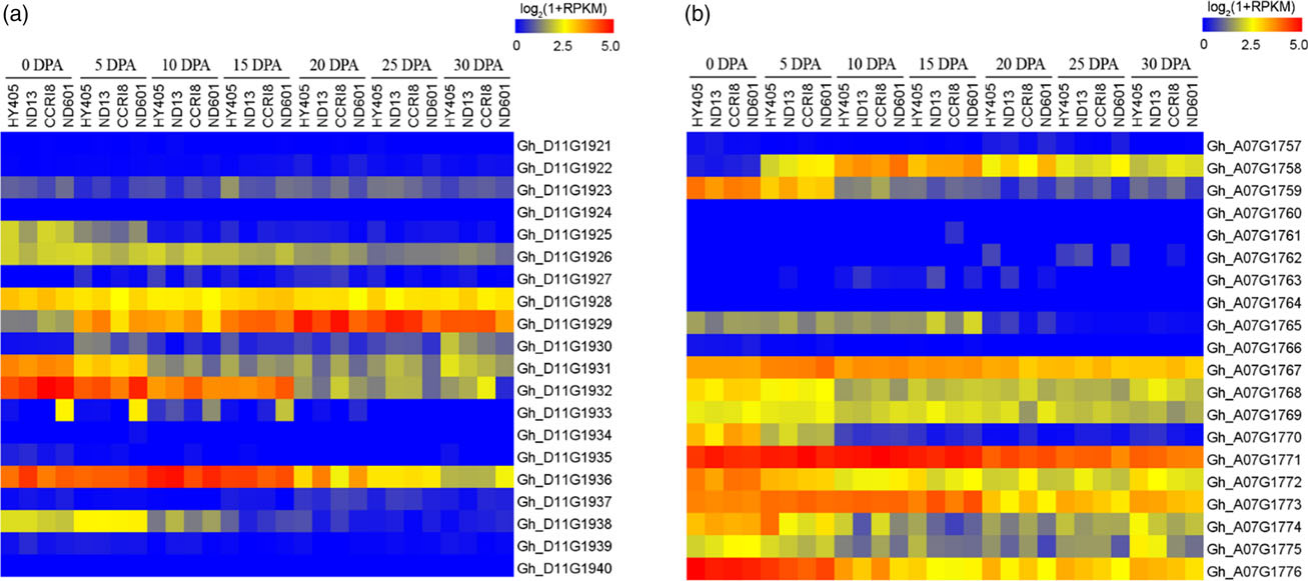
Expression pattern of candidate genes in the peak regions of Dt11 and At07. (a) Heat map of the expression of 20 genes associated with fibre length in Dt11 among four upland cotton cultivars. (b) Heat map of the expression of 20 genes in At07 among four upland cotton cultivars.

In addition, we verified 20 associated genes in FL and 20 genes in FS surrounding the peak SNPs in Dt11 and At07 based on expression (Figure 8a, b). Three genes (*Gh_D11G1926*,* Gh_D11G1928* and *Gh_D11G1929*) in Dt11 for FL contained a significant SNP locus (Figure 4b) and had higher expression level, especially *Gh_D11G1929* (Figure 8a). This gene was highly expressed at all time points of fibre development (Figure 6a, b; Figure 8a). There were seven genes not expressed during fibre development. Most of the genes (except the five genes that were not expressed) associated with the seven SNPs in At07 for FS showed high expression levels (Figure 8b). The two loci i02034Gh and i02037Gh were located inside gene *Gh_A07G1768* and *Gh_A07G1769*, respectively (Figure 5b). Moreover, there were three SNPs near the two genes (Figure 5b) that were steadily expressed from 0 to 30 DPA (Figure 8b). These significant SNPs had an effect on the neighbouring genes. One of these genes, *Gh_A07G1768*, annotated as an unknown protein, may be a novel gene for fibre development (Table S3). In addition, the expression of gene *Gh_A07G1758* near locus i39753Gh remained high (Figure 6a, b; Figure 8b). These data suggested that the above candidate genes play important roles in different stages of fibre development. Additionally, among the 26 significantly expressed genes in the haplotypes, there were three known and 23 not reported genes in cotton (Table S3).

## Discussion

In this study, we first performed a genome‐wide association analysis of fibre quality traits based on the CottonSNP63K array with a large number of natural accessions of *G. hirsutum*. This study uncovered numerous loci underlying variation in fibre quality traits and identified a set of candidate genes that could be exploited to alter fibre development to improve upland cotton cultivars.

By phenotyping the 719 cotton accessions in various environments, relatively abundant variation and significant differences among genotypes and environments were observed for the fibre quality traits (Table 1; Table S2), which showed higher diversity compared with previous reports in cotton (Jamshed *et al*., 2016; Shen *et al*., 2005; Yang *et al*., 2014b). The panel was classified into two groups based on molecular analyses, with 68 unique SNPs in G1 and 360 unique SNPs in G2 (Figure 1c, d); however, they were not completely consistent with geographical sources due to the wide adaptation of cotton, as in previous studies (Tyagi *et al*., 2014; Wang *et al*., 2016). The differences in polymorphic SNPs likely reflected the history of extensively interspecific crosses between diverse accessions of the two groups, resulting in introgression among germplasms. Therefore, it was difficult to fully classify the cotton accessions according to geographical source (Li *et al*., 2014; Xu *et al*., 2016). In addition, LD decay limits the mapping resolution of GWAS. Our study found that the LD decay was 0.82 Mb, which provided a more accurate reference for selecting candidate genes than previous studies using SSRs (Abdurakhmonov *et al*., 2008; Nie *et al*., 2016). Taking account of the greater continuous phenotypic variation among the tested fibre quality traits, our not highly structured association panel is applicable for performing GWAS.

Historically, QTL mapping has been utilized to identify and map causative genomic locations controlling fibre quality using biparental populations. Hundreds of QTLs have been located in intraspecific and interspecific populations of cotton (Said *et al*., 2015), but this method has rarely led to candidate gene isolation because it only captures limited allelic diversity existing in two parental lines. In contrast to traditional linkage mapping, GWA mapping does not require biparental crosses between individuals and provides greater precision in the localization of QTLs. This approach has been widely used for various traits in other crops, including morphological traits (Crowell *et al*., 2016; Li *et al*., 2014; Meijon *et al*., 2014; Porth *et al*., 2013), disease resistance (Dimkpa *et al*., 2015; Wang *et al*., 2012; Wen *et al*., 2014), environmental adaptation (Famoso *et al*., 2011; Nagel *et al*., 2015; Wu *et al*., 2015; Yang *et al*., 2014a; Zhang *et al*., 2015a) and flowering time (Harsh *et al*., 2015; Stracke *et al*., 2009; Xu *et al*., 2016). In cotton, efforts have been made to detect QTLs using GWAS with SSR markers in recent years (Abdurakhmonov *et al*., 2008; Cai *et al*., 2014; Nie *et al*., 2016; Zeng *et al*., 2009). These GWAS results, although conducted with few markers and samples, are important for fibre improvement. However, limitations of mapping resolution and genome coverage are inevitable. Therefore, in this study, the genetic factors controlling fibre quality discovered by GWA mapping with more SNP markers and cotton accessions could provide more precise insight into fibre development.

A combination analysis of our GWAS and transcriptome sequencing data revealed 163 and 120 significantly expressed candidate genes in FL and FS, respectively. Two of these genes, *Gh_A07G1758* and *Gh_D11G1929*, were associated with fibre length and strength. *Gh_A07G1758* on At07 is homologous to *AT4G17170* (RAB GTPase B1C) and *Gh_D11G1929* on Dt11 codes for KIP‐related protein 6 (KRP6) in *Arabidopsis*. The Rab family encodes key factors of vesicle‐targeting specificity proteins in *A. thaliana* (Rutherford and Moore, 2002). A plant Rab GTPase, RabA4b, was proposed to regulate membrane trafficking in cells (Preuss *et al*., 2004). Another plant‐unique RAB5 protein, ARA6, was demonstrated to have a functional link between a specific RAB acting in the endosomal trafficking pathway and a specific SNARE complex in *Arabidopsis thaliana* (Ebine *et al*., 2011). Complexes of SNARE proteins mediate intracellular membrane fusion between vesicles and organelles to facilitate transport cargo proteins in plant cells (Baker and Hughson, 2016). These results indicated that *Gh_A07G1758* could play a key role in the formation of cotton fibre, as indicated by a QTL (Zhang *et al*., 2011) near *Gh_A07G1758* found in previous research. The detailed mechanism of this gene should be further investigated. In addition, we identified a gene, *Gh_D06G0002*, encoding a SNARE‐like superfamily protein homologue, which was not previously reported in cotton. *Gh_D11G1929* is a homologue of *A. thaliana KRP6*, which is a cyclin‐dependent kinase inhibitor. In *Arabidopsis thaliana*,* KRP6* overexpression accelerated entry into mitosis, but delayed mitotic progression (Vieira *et al*., 2014). The regulator *KRP6* partially repressed GA‐dependent activation of the cell cycle during germination (Nieuwland *et al*., 2016). However, analysis of this gene in cotton fibre development has not been reported.

Many genes participating in nucleotide sugar metabolism are important in fibre cells. An identified gene, *Gh_D03G0294*, homologous to Arabidopsis xyloglucan endotransglucosylase/hydrolase (XTH), made plant cells undergo cell expansion, acting as a cell wall‐loosening enzyme (Van Sandt *et al*., 2007). *GhXTH1* was the predominant *XTH* in elongating fibres, and its expression limited cotton fibre elongation (Lee *et al*., 2010). Another gene, *Gh_D05G1451*, homologous to Arabidopsis trehalose‐6‐phosphate synthase (TPS), was implicated in the regulation of sugar metabolism/embryo development (Eastmond *et al*., 2002). Arabidopsis *TPS1* may play a major role in coordinating cell wall biosynthesis and cell division in cellular metabolism (Gomez *et al*., 2006). These two genes play different roles during fibre development based on their expression patterns in cotton (Figure 6); however, the functions of these genes in cotton remain to be elucidated.

In conclusion, we genotyped 719 upland cotton accessions using the CottonSNP63K array for the first time and identified 46 SNPs significantly associated with fibre quality traits across eight environments and a number of novel genes, including 19 promising genes, of which ten were not reported in cotton, for FL and FS by GWA mapping. The identified genetic variation and candidate genes deepen our understanding of the molecular mechanisms underlying cotton fibre development. The validated accessions with excellent haplotypes are valuable breeding materials to improve cultivars. Our study provides a new resource for the improvement of cotton fibre quality through biotechnology‐assisted selection in future breeding efforts.

## Experimental procedures

### Cotton accessions and field experiments

A collection of 719 upland cotton germplasms (*Gossypium hirsutum* L.) was used for the association analysis in this study. These accessions were from different countries, 588 were collected from China and 131 were from other countries (Table S1). The 719 accessions were grown in eight natural environments in a randomized complete block design at Baoding (115°47′N, 38°87′E), Hejian (116°13′N, 38°42′E), Xinji (115°12′N, 37°54′E) and Qingxian (116°91′N, 38°65′E) in Hebei Province and Yacheng (109°20′N, 18°38′E) in Hainan Province in 2014, denoted 14BD (E1), 14HJ (E2), 14XJ (E3), 14QX (E4) and 14HN (E5), respectively, and Xinji and Qingxian in Hebei Province and Yacheng in Hainan Province in 2015, denoted 15XJ (E6), 15QX (E7) and 15HN (E8), respectively. Two replicates were performed for each accession in five locations in 2014, and three replicates were performed for the three locations in 2015. Briefly, one row of each accession was planted for each replicate, with 20–22 plants per row, 30–35 cm between plants within rows and 80 cm between rows.

### Phenotypic evaluation and statistical analyses

When mature, 20 naturally open bolls from the central part of the plants from each accession were hand harvested at each location and ginned. Fibre samples were sent to the Supervision and Testing Center of Cotton Quality, Ministry of Agriculture of China in Anyang, Henan Province for fibre property determination. Fibre quality traits, including the upper half mean fibre length (FL, mm), fibre strength (FS, cN/tex), fibre micronaire (FM), fibre uniformity (FU, %) and fibre elongation (FE, %), were measured using a high volume instrument (HVI). Statistical analyses, Pearson correlation between traits and significance analyses were conducted using SPSS 22.0 software. Differences were tested for significance at the 1% probability level.

### Genotyping and SNP marker screening

Genomic DNA of each accession was extracted from young leaf tissues for genotyping using a modified CTAB method (Zhang and Stewart, 2000). A CottonSNP63K array containing 63 058 SNPs (Hulse‐Kemp *et al*., 2015), which was recently developed by an international cotton SNP consortium, was applied to genotype the 719 accessions using the Illumina Infinium platform according to the manufacturer's protocol. All the SNP data were clustered and selectively analysed by Illumina GenomeStudio genotyping software. The SNP data set was further filtered with a calling rate < 0.85 and MAF < 0.05. For the physical localization of SNP markers, the probe sequences of the SNPs were used to perform a local BLAST (Altschul *et al*., 1990) search against the *G. hirsutum* TM‐1 reference genome (Zhang *et al*., 2015b). SNPs that could not be assigned to a *G. hirsutum* chromosome were excluded from further analysis.

### Population structure and association mapping analysis

STRUCTURE 2.3.4 software (Evanno *et al*., 2005) was used to estimate the genetic structure of the population consisting of 719 accessions based on polymorphic SNPs. The numbers of hypothetical groups ranged from K = 1 to 10, using an admixture model with ten independent runs of 10 000 burn‐in time and 10 000 MCMC (Markov chain Monte Carlo) replication number. The output from STRUCTURE was analysed for the delta K value (ΔK) in STRUCTURE HARVESTER (Earl and vonHoldt, 2011). The optimal K value was determined by the log probability of LnP(K) and delta K based on the rate of change of LnP(K) between successive K. The Q matrix was derived for the subsequent association mapping which was the result of the integration of the cluster membership coefficient of replicate runs from STRUCTURE using CLUMPP software (Jakobsson and Rosenberg, 2007). Principal component analysis (PCA) using GCTA software (Yang *et al*., 2011) was used to assess the population structure. PowerMarker version 3.25 (Liu and Muse, 2005) was used to construct a NJ phylogenetic tree by calculating Nei's genetic distance among individuals.

For the genome‐wide association analysis, TASSEL 3.0 software (Bradbury *et al*., 2007) was used to determine the association between SNPs and phenotypic traits using a mixed linear model (MLM) (Zhang *et al*., 2010). The Q matrix from STRUCTURE and the kinship calculated with TASSEL 3.0 were included as fixed and random effects, respectively. The LD parameter (r^2^) between pairwise SNPs (MAF > 0.05) was estimated using TASSEL 3.0 software. The significance of the associations between SNPs and traits was based on the threshold of the Bonferroni correction for multiple tests (1/*n*), where *n* was the total number of SNPs used in the association analysis.

### Transcriptome sequencing

Two high‐quality upland cotton cultivars (HY405 and ND13) and two normal‐quality upland cotton cultivars (CCRI8 and ND601) were grown in the field of Baoding, China, in 2014. For the cotton fibre samples, bolls were collected at 0, 5, 10, 15, 20, 25 and 30 DPA. Samples from different plants were pooled. Total RNA was extracted from these samples using the EASY spin Plant RNA kit (Aidlab, Beijing, China). The qualified RNA was used for sequencing analysis using TopHat v2.0 on a HiSeq 2500 platform at the Novogene Bioinformatics Institute, Beijing, China.

## Conflict of interest

The authors declare no conflict of interest.

## Supporting information


**Figure S1.** Correlation analysis between five traits related to fibre quality.
**Figure S2.** Manhattan plots showing the GWAS for FL in eight environments.
**Figure S3.** Manhattan plots showing the GWAS for FS in eight environments.
**Figure S4.** Manhattan plots showing the GWAS for FM in eight environments.
**Figure S5.** Manhattan plots showing the GWAS for FU in eight environments.
**Figure S6.** Manhattan plots showing the GWAS for FE in eight environments.
**Figure S7.** Boxplots depicting the genetic effects of SNPs with significant associations with fibre length in Dt11.
**Figure S8.** Boxplots depicting the genetic effects of SNPs with significant associations with fibre length and strength in At07.
**Figure S9.** Expression of all candidate genes related to fibre length and strength.

**Table S1.** List of 719 upland cotton accessions used for association mapping.
**Table S2.** Analysis of variance (ANOVA) results of the fibre quality traits.
**Table S3.** List of 612 candidate genes with fibre quality traits.
**Table S4.** KEGG analysis of all candidate genes.
**Table S5.** Phenotypes of the fibre length and strength of 47 upland cotton accessions belonging to Hap3.
