# Supplementary material for: Genome‐wide association study discovered genetic variation and candidate genes of fibre quality traits in Gossypium hirsutum L
Source: Plant Biotechnol J. 2017 Mar 7;15(8):982–96. doi: 10.1111/pbi.12693 (PMC5506648; doi:10.1111/pbi.12693)
Supplement: Supplementary file 1 — Figure S1. Correlation analysis between five traits related to fibre quality. Figure S2. Manhattan plots showing the GWAS for FL in eight environments. Figure S3. Manhattan plots showing the GWAS for FS in eight environments. Figure S4. Manhattan plots showing the GWAS for FM in eight environments. Figure S5. Manhattan plots showing the GWAS for FU in eight environments. Figure S6. Manhattan plots showing the GWAS for FE in eight environments. Figure S7. Boxplots depicting the genetic effects of SNPs with significant associations with fibre length in Dt11. Figure S8. Boxplots depicting the genetic effects of SNPs with significant associations with fibre length and strength in At07. Figure S9. Expression of all candidate genes related to fibre length and strength. [file PBI-15-982-s002.docx]

**Supporting Information**


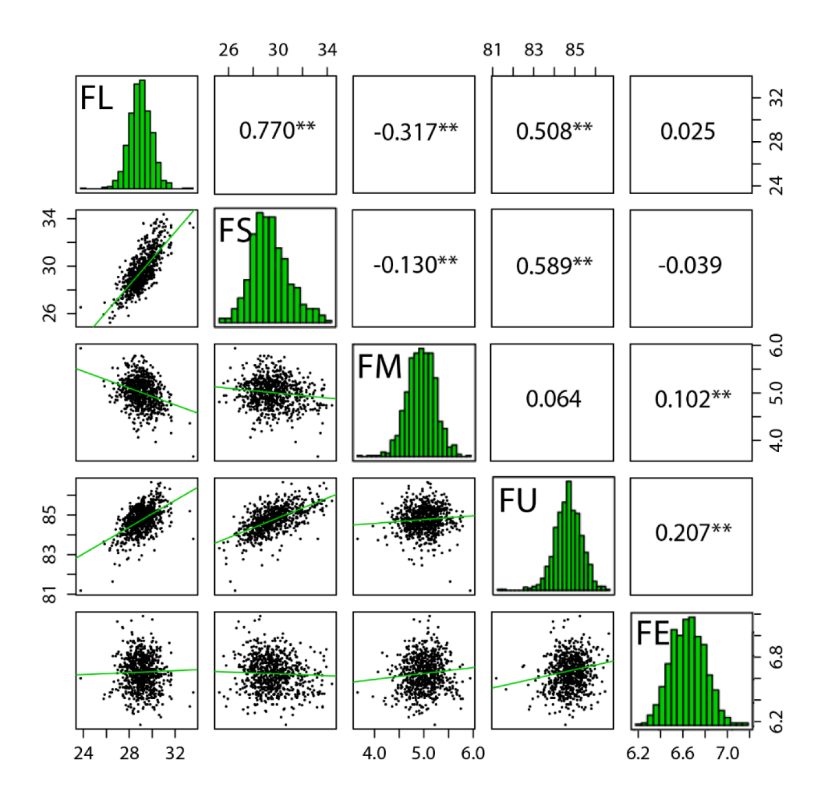


**Figure S1.** Correlation analysis between five traits related to fibre quality. FL, Fibre Length; FS, Fibre Strength; FM, Fibre Micronaire; FU, Fibre Uniformity; FE, Fibre Elongation. ** representing significance at P < 0.01 level (two-tailed).

**
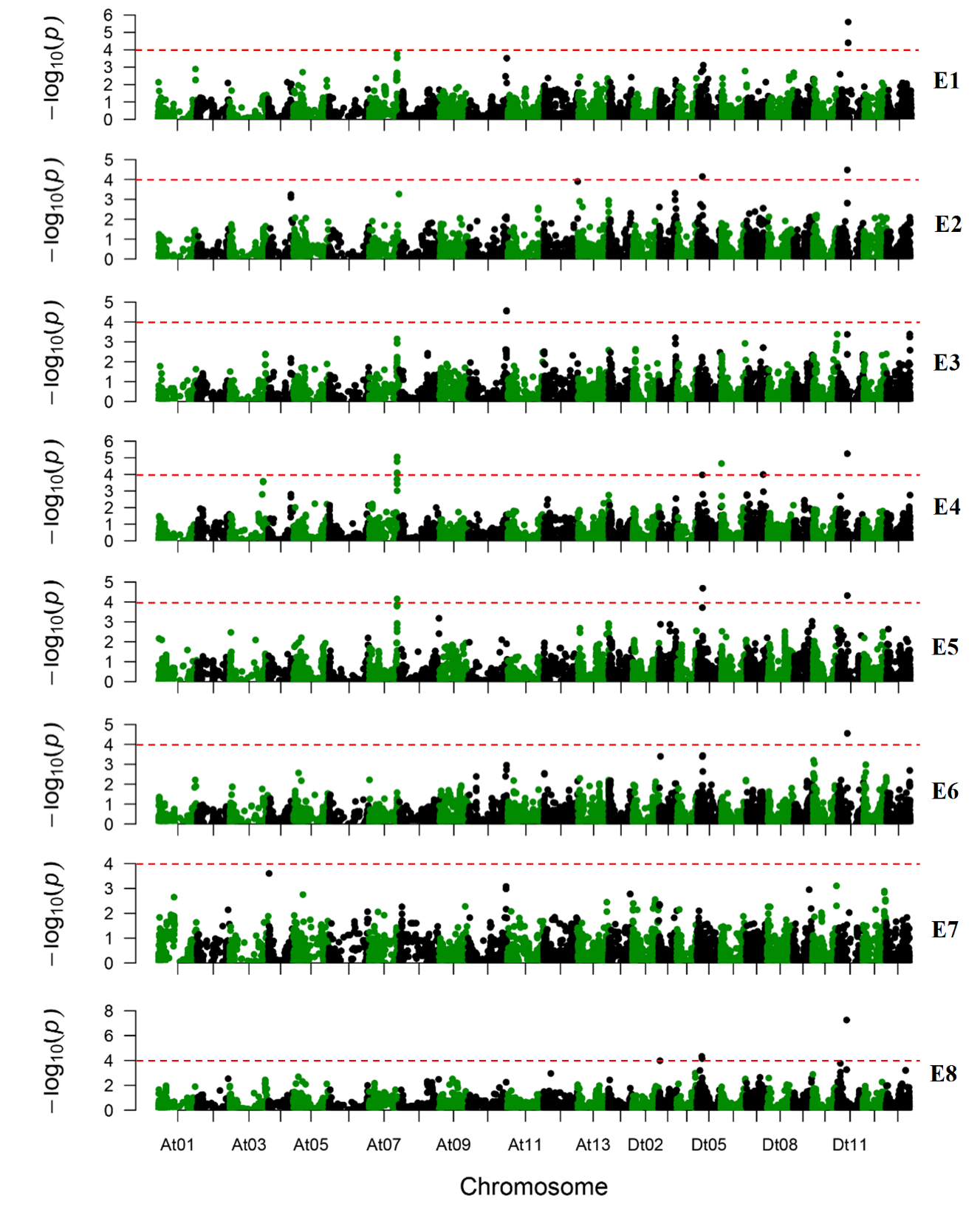
**

**Figure S2.** Manhattan plots showing the GWAS for fibre length in eight environments. The dashed horizontal line represents the significance threshold (P < 10^-3.97^).

**
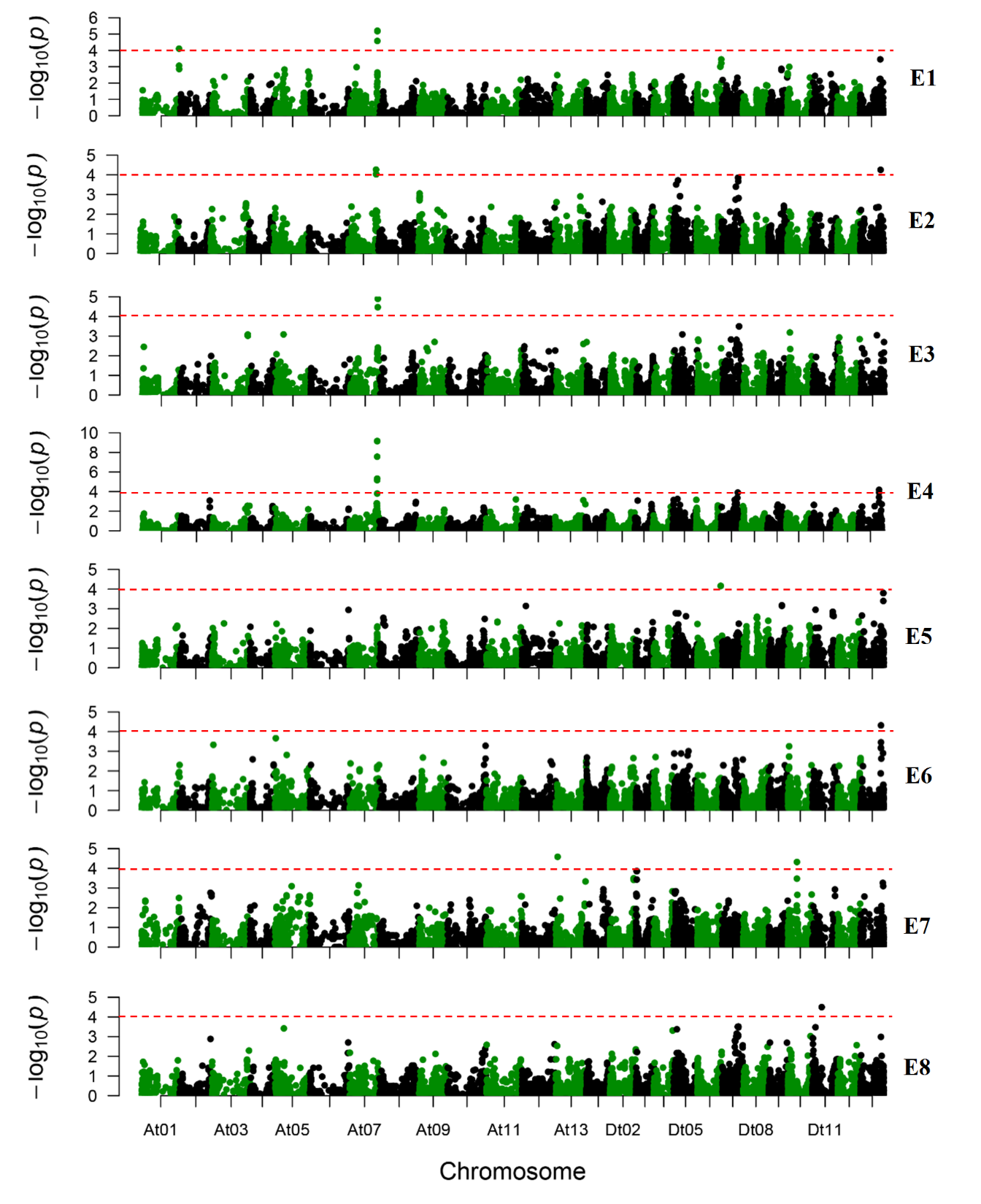
**

**Figure S3.** Manhattan plots showing the GWAS for fibre strength in eight environments. The dashed horizontal line represents the significance threshold (P < 10^-3.97^).

**
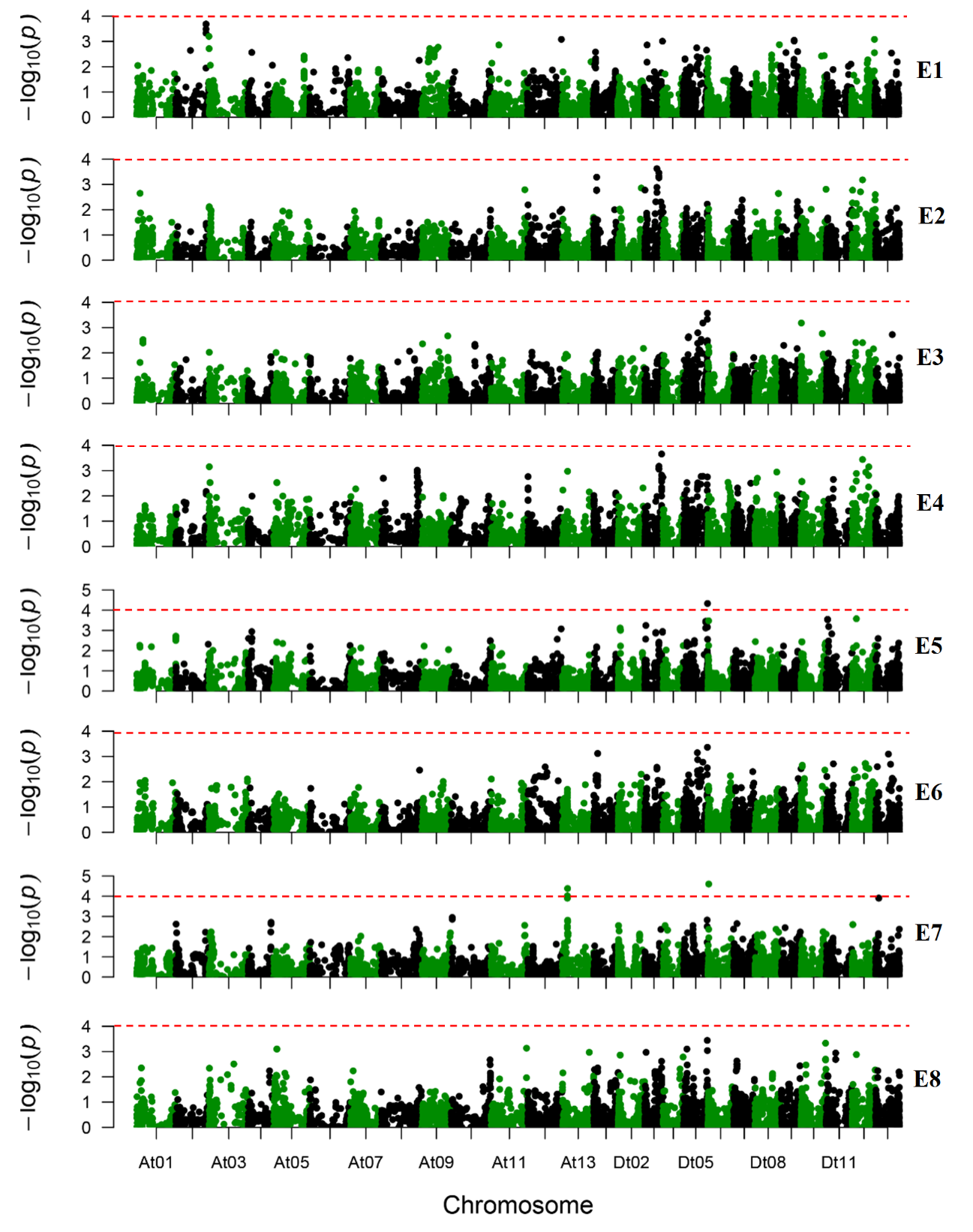
**

**Figure S4.** Manhattan plots showing the GWAS for fibre micronaire in eight environments. The dashed horizontal line represents the significance threshold (P < 10^-3.97^).

**
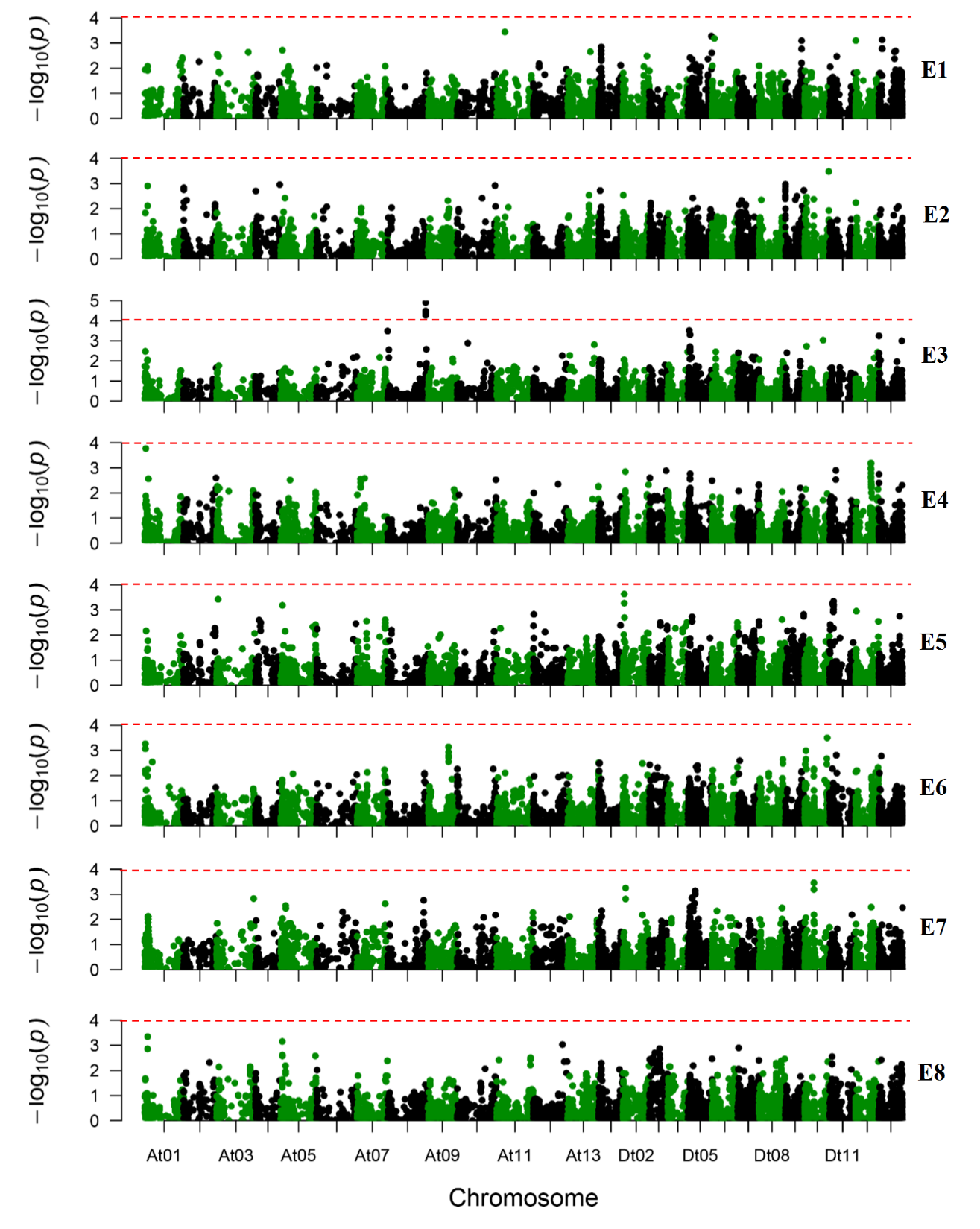
**

**Figure S5.** Manhattan plots showing the GWAS for fibre uniformity in eight environments. The dashed horizontal line represents the significance threshold (P < 10^-3.97^).

**
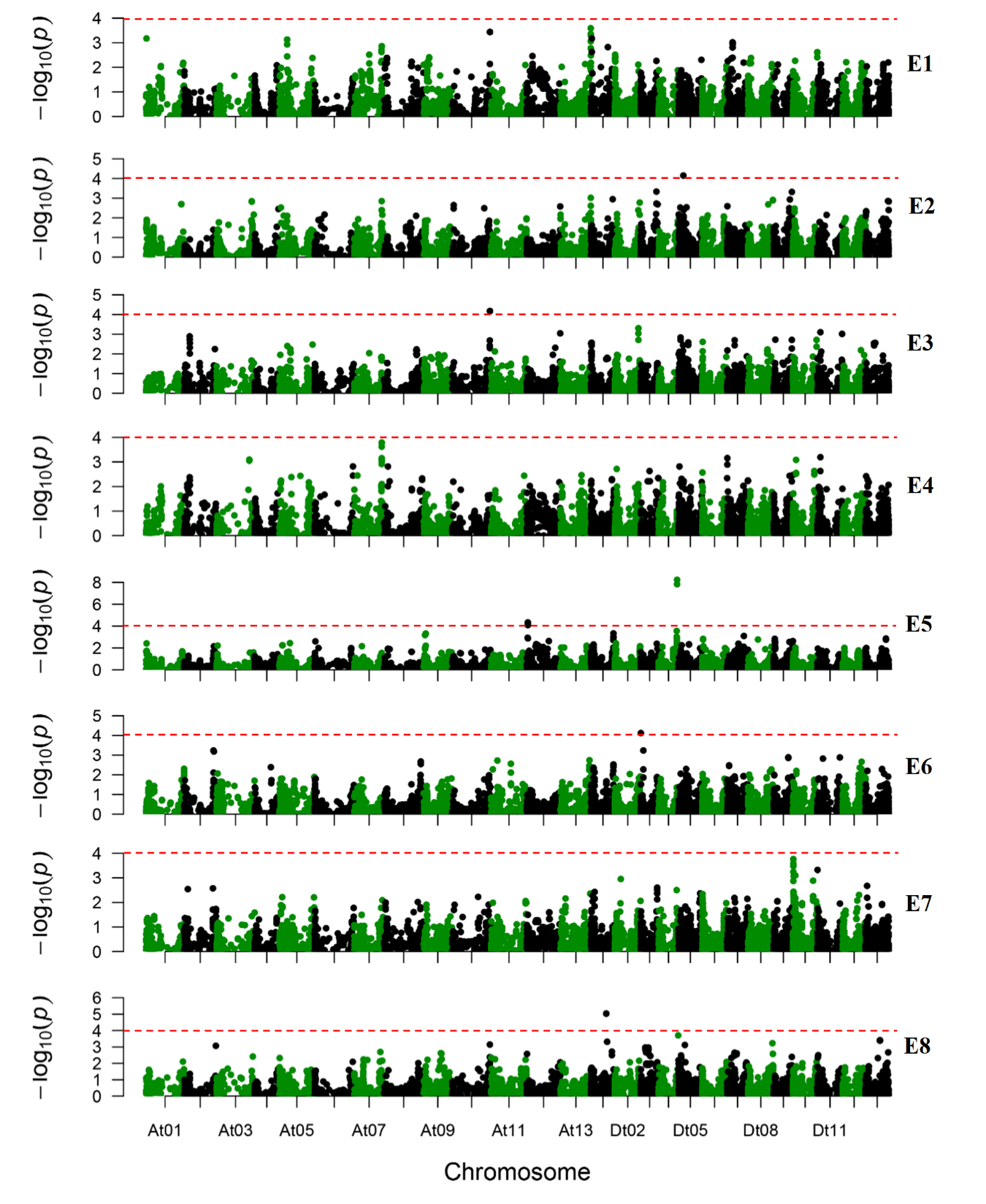
**

**Figure S6.** Manhattan plots showing the GWAS for fibre elongation in eight environments. The dashed horizontal line represents the significance threshold (P < 10^-3.97^).

**
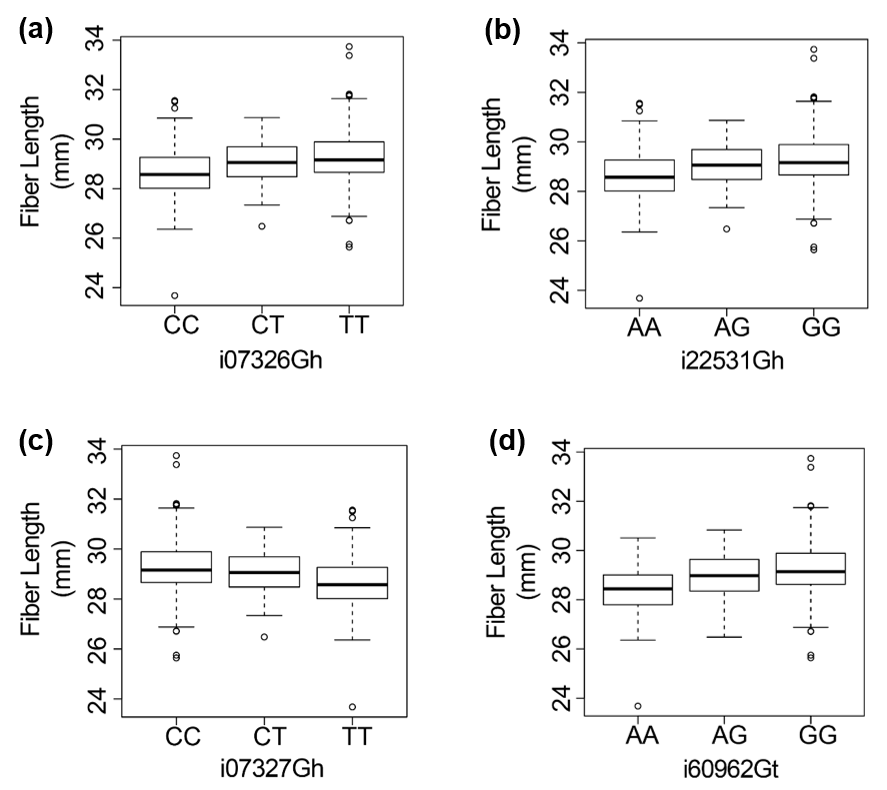
**

**Figure S7.** Boxplots depicting the genetic effects of SNPs with significant associations with fibre length in Dt11. (a) Boxplot diagram depicting the genetic effect of an SNP i07326Gh associated with fibre length; (b) Boxplot diagram depicting the genetic effect of an SNP i22531Gh associated with fibre length; (c) Boxplot diagram depicting the genetic effect of an SNP i07327Gh associated with fibre length; (d) Boxplot diagram depicting the genetic effect of an SNP i60962Gt associated with fibre length. The box shows the lower quartile, and the median and upper quartile values, and the whiskers show the range of the phenotypic variation in the population.

**
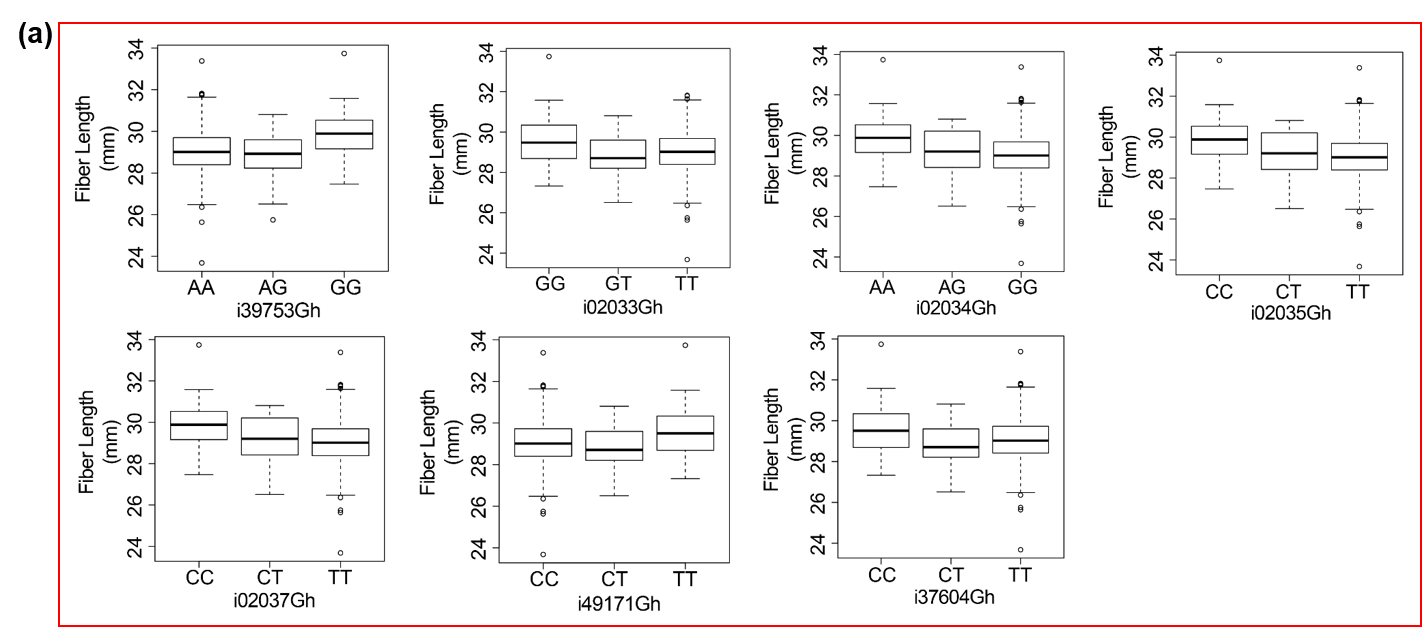
** **
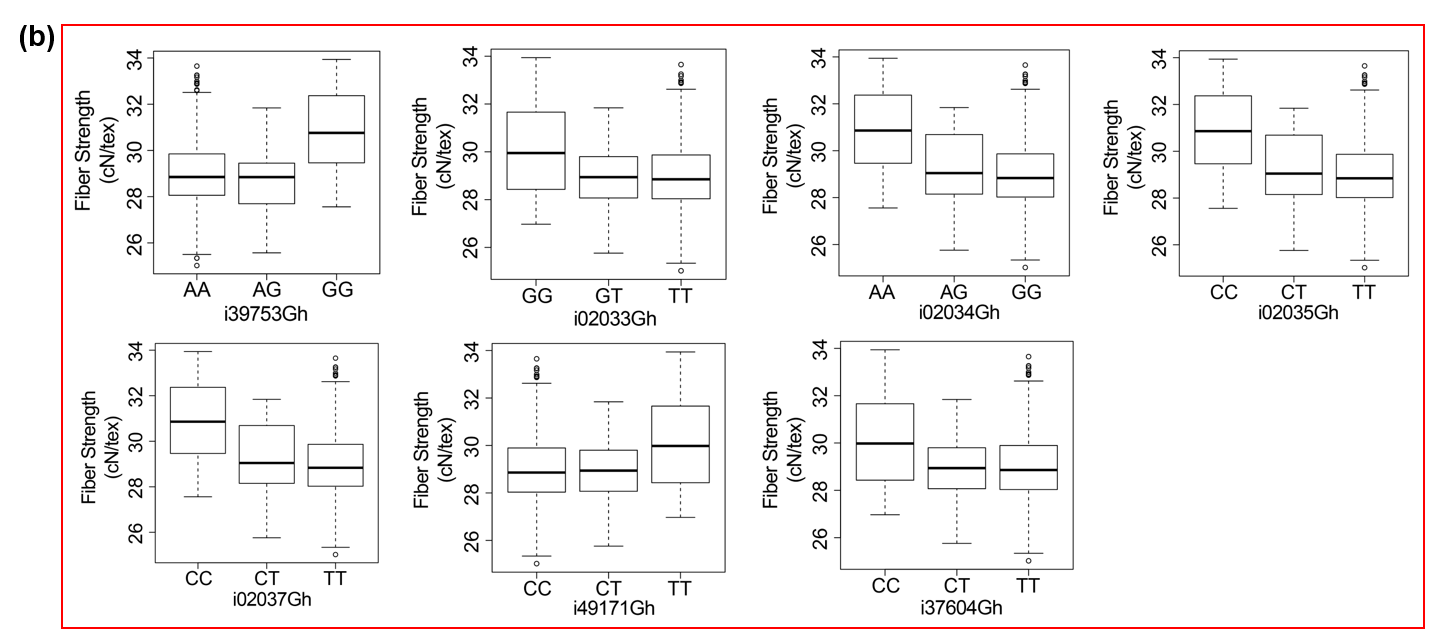
**

**Figure S8.** Boxplots depicting the genetic effects of SNPs with significant associations with fibre length and strength in At07. (a) Boxplot diagram depicting the genetic effect of seven significant SNPs to fibre length. (b) Boxplot diagram depicting the genetic effect of seven significant SNPs to fibre strength. The box shows the lower quartile, and the median and upper quartile values, and the whiskers show the range of the phenotypic variation in the population.

**(a)**

**(b)**


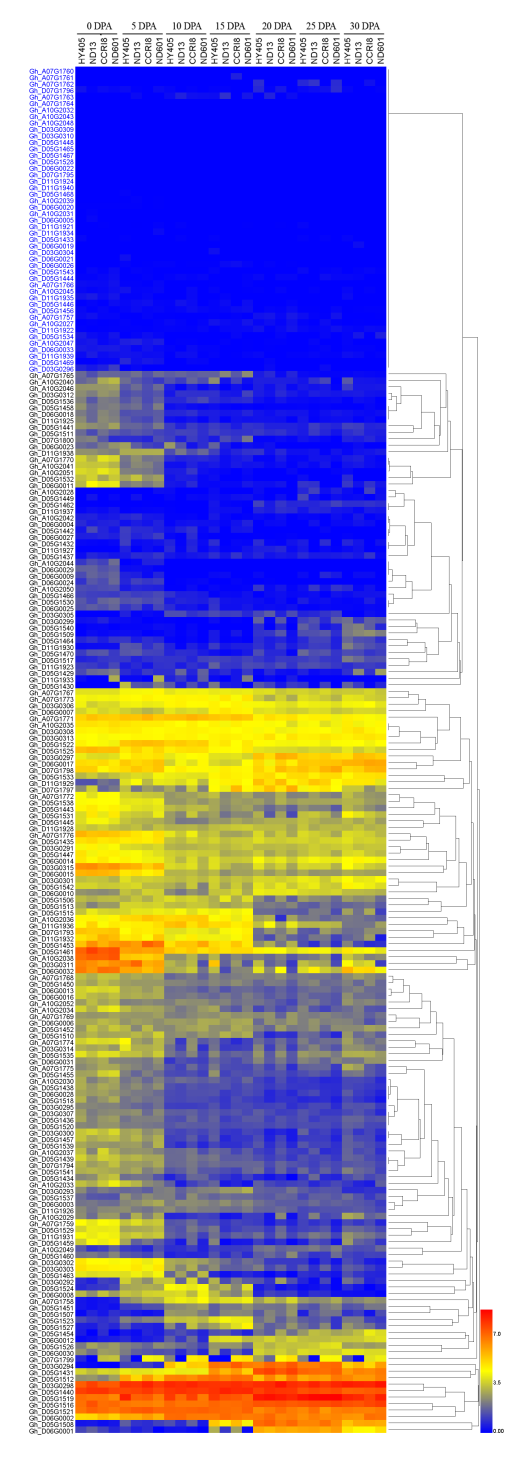
 **
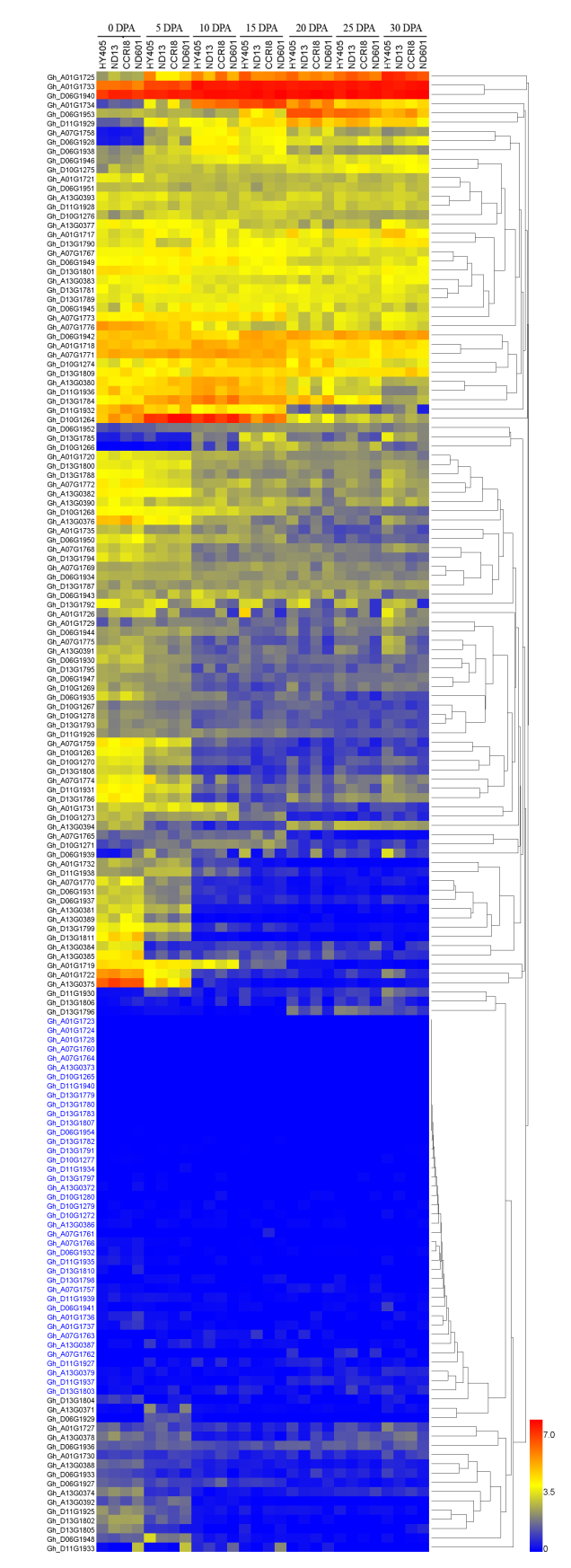
**

**Figure S9.** Expression of all candidate genes related to fibre length and strength. (a) Heat map of 212 candidate genes of fibre length. (b) Heap map of 161 candidate genes of fibre strength.
